# Phenotypic Convergence in Genetically Distinct Lineages of a *Rhinolophus* Species Complex (Mammalia, Chiroptera)

**DOI:** 10.1371/journal.pone.0082614

**Published:** 2013-12-03

**Authors:** David S. Jacobs, Hassan Babiker, Anna Bastian, Teresa Kearney, Rowen van Eeden, Jacqueline M. Bishop

**Affiliations:** 1 Department of Biological Sciences, University of Cape Town, Cape Town, South Africa; 2 Ditsong Museum of Natural History, Pretoria, South Africa; Smithsonian Conservation Biology Institute, United States of America

## Abstract

Phenotypes of distantly related species may converge through adaptation to similar habitats and/or because they share biological constraints that limit the phenotypic variants produced. A common theme in bats is the sympatric occurrence of cryptic species that are convergent in morphology but divergent in echolocation frequency, suggesting that echolocation may facilitate niche partitioning, reducing competition. If so, allopatric populations freed from competition, could converge in both morphology and echolocation provided they occupy similar niches or share biological constraints. We investigated the evolutionary history of a widely distributed African horseshoe bat, *Rhinolophus darlingi*, in the context of phenotypic convergence. We used phylogenetic inference to identify and date lineage divergence together with phenotypic comparisons and ecological niche modelling to identify morphological and geographical correlates of those lineages. Our results indicate that *R. darlingi* is paraphyletic, the eastern and western parts of its distribution forming two distinct non-sister lineages that diverged ~9.7 Mya. We retain *R. darlingi* for the eastern lineage and argue that the western lineage, currently the sub-species *R*. *d. damarensis*, should be elevated to full species status. *R. damarensis* comprises two lineages that diverged ~5 Mya. Our findings concur with patterns of divergence of other co-distributed taxa which are associated with increased regional aridification between 7-5 Mya suggesting possible vicariant evolution. The morphology and echolocation calls of *R. darlingi* and *R. damarensis* are convergent despite occupying different biomes. This suggests that adaptation to similar habitats is not responsible for the convergence. Furthermore, *R. darlingi* forms part of a clade comprising species that are bigger and echolocate at lower frequencies than *R. darlingi*, suggesting that biological constraints are unlikely to have influenced the convergence. Instead, the striking similarity in morphology and sensory biology are probably the result of neutral evolutionary processes, resulting in the independent evolution of similar phenotypes.

## Introduction

Geographic variation in fitness-enhancing traits is often attributed to local selection on populations occupying diverse habitats [1–3]. These traits can however also be highly convergent even in populations occupying different habitats. Phenotypic convergence by organisms in different habitats has often been attributed to adaptation to similar ecological niches [4,5]. Such convergence has often been used as evidence for adaptation but recently the causal relationship between convergence and adaptation has been questioned [5] with the realization that other factors such as random genetic drift can also lead to convergence [6]. This can make the interpretation of convergence and its biological significance problematical. 

For example, when species designations are predominantly informed by phenotypic traits, strong convergence in such traits can mask not only the existence of cryptic species but also the causes of speciation. Molecular tools have been instrumental in revealing cryptic biodiversity in such evolutionarily diverse groups as birds [7], mammals [8], echinoderms [9], anurans [10], fish [4,11] and arthropods [12], with important consequences for regional biodiversity planning [13]. 

Many phenotypically cryptic species uncovered so far co-occur e.g. [4,14] raising questions about how they can co-exist given their convergent phenotypes. Competition theory holds that ecologically similar co-existing species must partition their resources [15,16]. It is possible that very small differences in morphology, for example, can result in the use of different ecological niche space, allowing similar species to co-exist e.g. [17,18]. Alternatively, as has been discovered in bats, subtle niche partitioning amongst morphologically convergent species can be mediated by differences in their sensory biology [19–21]. 

Morphology in bats can be highly convergent [22–24] because many of them occupy similar ecological niches [25,26]. Consequently many species designations based on shared morphology and ecology have been found, through the use of molecular techniques, to consist of both divergent clades as well as closely related species complexes [22,27]. In recent years several morphologically cryptic bat species, confirmed by genetic analyses, have been uncovered largely as a result of differences in their sensory systems viz. the use of different echolocation call frequencies [19,20,28–31]. The evolution of highly convergent morphology in genetically distinct, yet sympatric, species with divergent echolocation frequencies is a recurrent theme in bats e.g. [19,20,24,29]. This has led to the suggestion that echolocation may be implicated in evolutionary diversification via partitioning of prey and/or habitat [32] or discrete frequency bands. Partitioning of the latter supposedly facilitates intraspecific communication [27,29,33-35] within areas where congenerics co-occur. Social information may be encoded in small intraspecific differences in frequency [29] such that divergence in sensory traits may permit the co-existence of species with similar morphology. Acoustic divergence in sympatry could be an example of character displacement under interspecific competition provided divergence occurred as a result of such competition [36,37]. If patterns of convergent morphology with divergent acoustic signals are the result of co-occurrence it might be expected that allopatric populations could converge in both morphology and acoustic signals provided they occupy similar ecological niches albeit in different habitats and/or selection for adaptation to local habitats is weak. Convergence in allopatry can also be facilitated by non-adaptive processes such as random genetic drift in combination with shared biological constraints. Constraints limit the variation that can be produced such that genetically divergent lineages converge upon the same phenotype even in the absence of selection [5]. 

Significant convergence in morphology and sensory systems is characteristic of the horseshoe bats (Rhinolophidae) throughout their distribution in the Palaearctic, Indo-Malay and Afrotropic ecoregions. All species are placed in a single genus (*Rhinolophus*) [38] and divided into two major phylogenetic clades defined by their geography: ‘African’ and ‘Oriental’ [39]. The African rhinolophids currently comprise 27 species [40] and at least one species with a wide geographic range has been found to include cryptic lineages [31]. 

Here we investigate the evolution of phenotypic convergence in allopatry in a widely distributed southern African bat species, Darling’s horseshoe bat, *Rhinolophus darlingi* Andersen, 1905. Convergence – the independent evolution of similar phenotypes amongst different evolutionary lineages [5] – predicts that two or more species under consideration would be more similar to each other than either is to their nearest common ancestor or to their closest relatives [5,6]. We test these predictions using phylogenetic comparisons on morphological , echolocation, and environmental data, sampling across the range of *R. darlingi* in southern Africa. *Rhinolophus darlingi* has a wide but clearly disjunct distribution in southern Africa where it spans the sub-continent from the mesic east to the xeric west across several biomes including woodland and grasslands in the east and arid savanna, Succulent- and Nama-Karoo, shrubland and desert in the west [41,42]. Several sub-species are currently recognised [43] and molecular evidence suggests that *R. darlingi* may be polytypic. Individuals in the western half of South Africa revealed within-region sequence divergence at cytochrome b of 2.5% compared to between region divergence with individuals from eastern South Africa and Swaziland of 10 - 15% [44]. We therefore also included several other rhinolophid species in our analyses to test monophyly within *R. darlingi* and to accurately establish the geographic boundaries of the lineages uncovered. 

## Materials and Methods

### Ethics statement

Research reported here was done in accordance with guidelines of the American Society of Mammalogists (Animal Care and Use Committee 1998). This research was approved by the Science Faculty Animal Ethics Committee of the University of Cape Town (2011/v6/DJ) and conducted under permits from the permitting authorities in the respective countries (Namibia – 1429/2010; Zimbabwe – 23(1)(C) (11) 25/2011; South Africa – AAA003-00030-0035; 1197/2008). Land accessed was publicly or privately owned and sometimes protected. At all times the necessary permission was obtained. We did not sample protected species.

### Sampling

**Figure 1:**
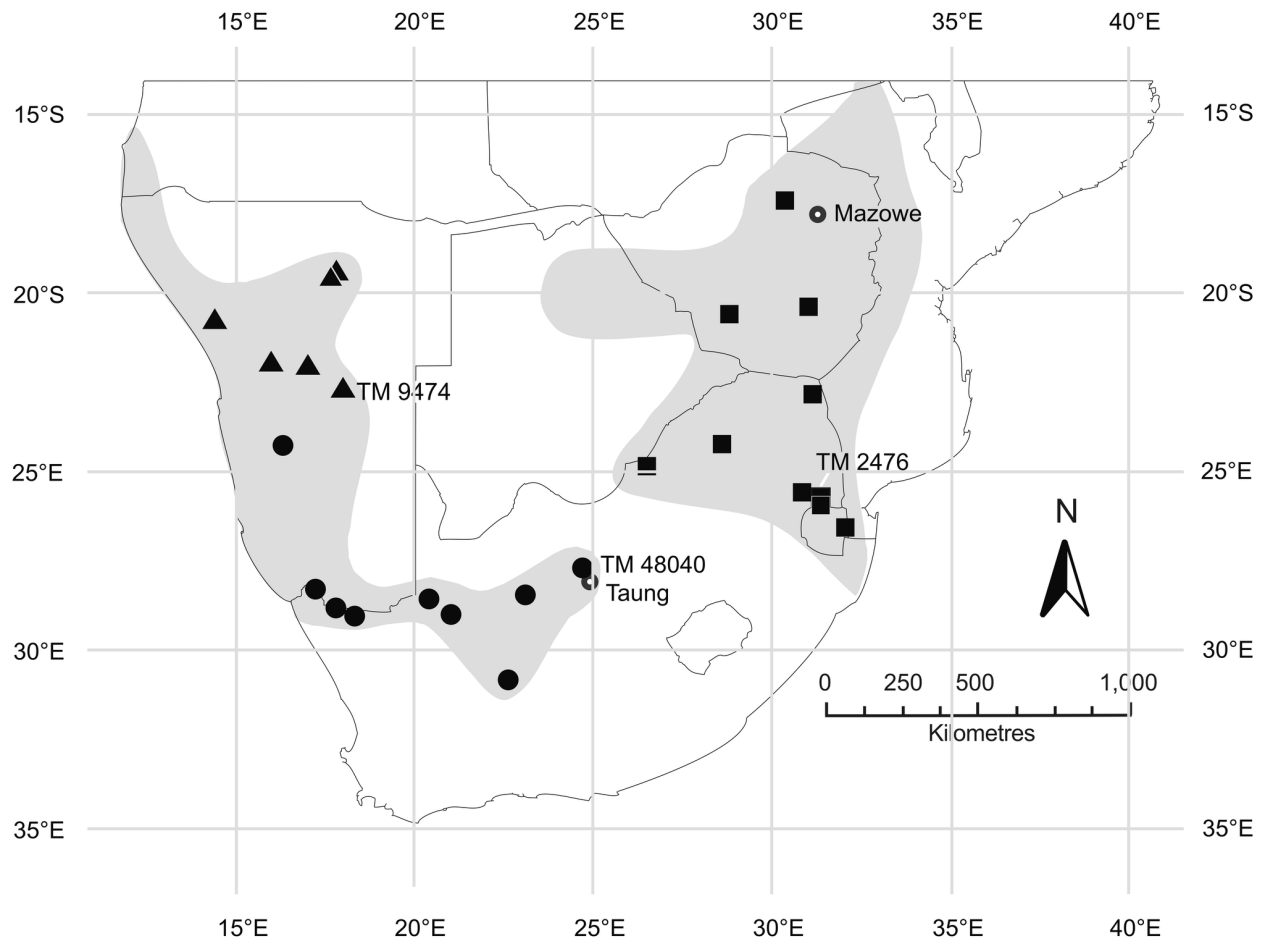
The distribution of *R. darlingi* (shaded areas) and sample localities (symbols). Squares = eastern *R. darlingi*; Circles (southern lineage) and triangles (northern lineage) = western *R. darlingi* (*R*. *damarensis*; Fig. 2). TM 9474 = holotype *R*. *d. damarensis* Namibia), TM 2476 = holotype of *R. d. barbetonensis* South Africa.

In the eastern part of its range *R. darlingi* is distributed from south-western Malawi, to north-western Mozambique, throughout Zimbabwe, north and eastern Botswana and the north-eastern region of South Africa including Swaziland. In the west it is currently known from north-west South Africa westward through Namibia, and just entering into south-west Angola (Figure 1; [42]). These geographically isolated distributions coincide with two of the currently recognized subspecies of *R. darlingi*, *R. darlingi darlingi* and *R. darlingi damarensis* [43]. The holotype (BMNH 1895.8.27.1) of *R*. *d. darlingi* comes from Mazowe, Mashonaland, Zimbabwe, while that of *R*. *d. damarensis* (TM 9474) was from Oserikari, Damaraland, Okahandja District, in Namibia (Figure 1). For this study, *R. darlingi* individuals were sampled together with several other rhinolophid species (Table S1 and Appendix S1 in File S1) at several sites in Namibia, South Africa and Zimbabwe from 2008 to 2012 (Figure 1). Species identifications were based on Monadjem et al. [42]. Wherever possible one male and one female were taken as vouchers from each site sampled. All other bats were released unharmed back into their roosts after data and tissue sample collection. We also obtained morphometric data and tissue samples from museum specimens of *R*. *d. darlingi* (See Table S2 and Appendix S1 in File S1). 

### Phylogenetic reconstruction and dating

We reconstructed the evolutionary relationships within and between *R. darlingi* (*sensu lato*) and several other rhinolophid species from Africa, Asia and Europe using the mitochondrial cytochrome b (cyt b) gene and the nuclear marker thyrotropin beta chain precursor (THY). 

#### Sample collection and DNA sequencing

Tissue samples for genetic analyses were taken from the tail membranes of captured bats using 3 mm diameter skin biopsy punches (stored in 70% ethanol), from pectoral muscle extracted prior to specimen preparation, or from dried skins of museum specimens. 

Total genomic DNA was extracted from samples using a DNeasy Blood & Tissue Kit (Qiagen; Appendix S2 and File S1) following the manufacturer’s instructions and stored at 4°C. Two dried skin samples from museum holotype specimens (TM 9474 [*R*. *d. damarensis*] and TM 2476 [*R. d. barbetonensis*]) were also used in the study. The samples were extracted using a QIAamp DNA Micro Kit (Qiagen) (see Appendix S2 in File S1). In total we sequenced 77 individuals for the nuclear marker and 48 for the mitochondrial marker. Sample details and collection site coordinates are reported in Table S2 in File S1 (Appendix S1 in File S1).

 DNA extractions were diluted where necessary to achieve a final concentration of approximately 40 ng/μl (using the NanoDrop 2000, Thermo Fisher Scientific Inc, USA). To amplify the cyt b region we used published primers L1472ag (5´-ATG ATA TGA AAA ACC ATC GTT G-3´) and H15915 (5´-TTT CCN TTT CTG GTT TAC AAG AC-3´) [45]. For the museum specimens the degraded DNA template required the use of a primerless PCR followed by a PCR in which multiple primer pairs were used to successfully amplify the target region. These multiple primers were specifically developed for this study (see Table S4 and Appendix S1 in File S1). The nuclear THY gene was amplified using published primers (5´-GGG TAT GTA GTT CAT CTT ACT TC-3´ and 5´-GGG ATC CTG GTA TTT CTA CAG TCT TG-3´) from [46] which were modified from those developed by [47]. Optimal concentrations and temperature conditions for PCRs are given in the Information (Appendix S2 in File S1). PCRs were performed on a GeneAmp 9700 thermocycler (Applied Biosystems) and checked under UV in 1% ethidium bromide agarose gel. Museum DNA template was successfully amplified using a combination of ‘primerless’ PCR reactions together with cold-start PCR design (Appendix S2 in File S1). PCR products were purified using a Wizard SV Gel and PCR Clean-up System (Promega) and sequenced in both directions using BigDye 3.1 chemistry on an ABI 3730 XL DNA Analyzer (Applied Biosystems at Macrogen Inc., South Korea). 

#### Sequence alignment and phylogenetic analyses

DNA sequences were edited in SeqMan II v6.00 (DNASTAR Inc. USA) and ChromasPro v1.5 (Technelysium PTY Ltd., Australia) and consensus sequences compiled from the reverse and forward sequence files. The minimum overlap of the two sequences was 150bp for cyt b and 350bp for the THY sequences. The final alignment for both sets of sequences comprised 32 unique cyt b sequences (Genbank accession numbers given in Table S3 and Appendix S1 in File S1) and 43 unique THY sequences (Genbank accession numbers given in Table S3 in File S1). The alignments were edited manually and translated into amino acids to verify the reading frame in MEGA4 [48]. The translated alignments had no gaps or stop codons. Sequences from *Hipposideros* spp. and other rhinolophids were downloaded from Genbank [49] and used as outgroups in the phylogenetic analysis (Table S3 and Appendix S1 in File S1). 

 Descriptive statistics for the two sequence data sets were calculated in MEGA4. Phylogenetic analysis was performed using Maximum Parsimony (MP), Maximum Likelihood (ML) and Bayesian inference methods. For MP and ML, analyses were carried out in PAUP 4.0b10 [50] using PaupUp graphical interface [51]. Tree reconstruction using Bayesian inference was carried out in MrBayes v3.1.2 [52]. We also estimated the time to most recent common ancestor (TMRCA) for a number of clades of interest using BEAST v1.7.4 [53]. (Details for all analyses are given in Supporting Information, Appendix S2).

### Echolocation

Echolocation calls were recorded from hand-held bats of several species of Rhinolophidae (Table S1 and Appendix S1 in File S1). Each bat was positioned 10 cm in front of an Avisoft Ultrasound Gate 416 (Avisoft Bioacoustics, Germany) microphone. Calls were recorded directly onto a notebook computer with Avisoft SasLab Pro software v5.1.23 using a sampling rate of 500,000 Hz. Recordings were slowed down by ten and were analysed using BatSound Pro v3.20, (Pettersson Elektronik AB, Uppsala, Sweden). The resting frequency (RF; Table S1 and Appendix S1 in File S1) – the frequency of highest energy in the constant component of the calls emitted when stationary – was determined for each bat from the fast Fourier transformation (FFT) power spectrum (size 1024 samples; frequency resolution 684 Hz). The resting frequency for each bat was taken as the average resting frequency of five to ten high-quality calls (calls in which the amplitude of the signal was at least three times higher than that of the background noise as displayed on the oscillogram). 

### Morphometric measurements from vouchers and museum specimens

#### Skull and post-cranial morphology

Skull and post-cranial parameters were measured to the nearest 0.01mm, using digital callipers, on each voucher specimen of *R. darlingi*. Several other rhinolophid species (*R. blasii*, *R. capensis* and *R. clivosus*) that are similar in size and/or use similar echolocation calls to *R. darlingi* (Table S1 and Appendix S1 in File S1 Supporting Information) and are thus likely to be confused with it were also measured. The parameters we measured were forearm length (FA); tail length (TL); tibia length (TB); ear length (EL); standard skull measurements (following Csorba et al., 2003) and standard wing elements (Table S1 and Appendix S1 in File S1 Supporting Information; [45]). 

#### Noseleafs

The shapes of noseleafs are highly variable even within bat lineages and are not useful as morphological correlates of genetic lineages [31]. We thus only compared the maximum width of noseleafs (NLW) amongst the genetic lineages identified in this study. Noseleaf width appears to have some value in characterizing species [42]. 

#### Baculum morphology

Bacula from several vouchers of *R. darlingi* captured in north western South Africa (Vioolsdrif on the Orange River) were removed and photographed for comparison with bacula from museum specimens of *R. darlingi* from central and eastern South Africa (Taung, North West Province and Gauteng Province, respectively) and from Swaziland (also in the eastern part of the sub-continent), as well as with those from *R. blasii*, *R. capensis* and *R. clivosus*. Preparation of the bacula followed the methods described by Goodman et al. [54]. Baculum images were taken with a Canon 350D camera attached via a LM-Scope Digital SLR Adapter to a Wild stereo microscope. Images were stacked using CombineZP software [55] for increased depth of field. Frames were aligned and balanced using the thorough routine, and stacked using pyramid weighted average. 

The following nine measurements were taken from each baculum using a microscope with an eyepiece graticule: greatest width of the base viewed in the dorsal plane (WB-d); greatest width of the base viewed in the lateral plane (WB-l); greatest baculum length (GBL); greatest width of the shaft (GW); length from the tip to the point of the greatest shaft width (LTW); narrowest width of the shaft (NW); length from the tip to the point of the narrowest shaft width (LTN); greatest length of the incision on the dorsal side of the base (BIL); and the height of the base, from the midpoint in the dorsal plane of the upper edge of the base to the most extended point at the edge of the base (BH).

### Statistical analyses of morphometric and echolocation data

A principal component analysis (PCA) on the combined external and skull data from vouchers and museum specimens was done to extract 24 independent and uncorrelated factors from the original set of 24 variables (Table S1 and Appendix S1 in File S1 Supporting Information; excluding NLW and PF) to meet the assumptions of discriminant function analysis (DFA). DFA was done on the factor scores of principal components with an Eigenvalue ≥ 1 (Kaiser’s criterion; [56]) to examine multivariate morphometric differences within and between *R. darlingi* and other cryptic rhinolophid species (Appendix S3 in File S1 Supporting Information). Prior to PCA, variables were standardized using the z-transformation in Statistica v10 (computed as Std. Score = (raw score – mean)/Std. deviation; [57]). Initial classification of individual bats in DFA followed the taxonomy in Monadjem et al. [42]. In addition, on the basis of evolutionary relationships revealed in our phylogenetic construction, the type localities and geographic ranges of the subspecies [42], we classified all *R. darlingi* specimens from the more arid western half of the subcontinent (i.e. Namibia, and central, north-western and south-western South Africa) as *R*. *d. damarensis* and those from the more mesic eastern half of the subcontinent (i.e. Zimbabwe and eastern and north-eastern South Africa including Swaziland) as *R*. *d. darlingi*. 

A multivariate analysis of variance (MANOVA) was done on nine measurements of 22 bacula from individuals of the various species included in the DFA (see Table S1 and Appendix S1 in File S1 Supporting Information).

**Figure 2:**
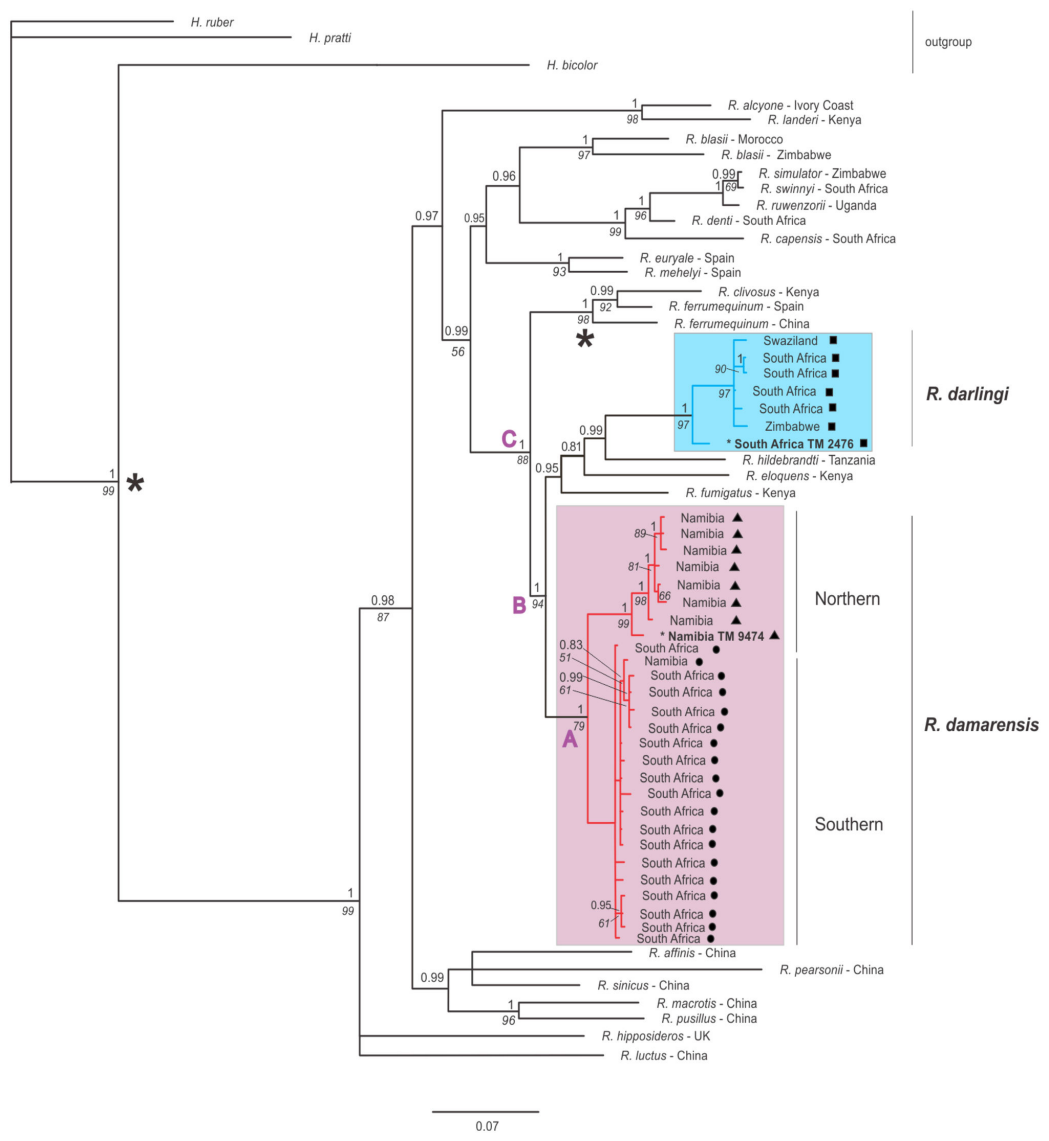
Bayesian consensus topology of *Rhinolophus* spp. based on cytochrome b. Values above nodes indicate Bayesian posterior probabilities (> 0.80) and below nodes report parsimony bootstrap support. Branch lengths are proportional to average number of substitutions per site. Divergence times (millions years ago, Mya) were estimated for three nodes of interest: node A = 4.8 Mya (95% HPD; 2.69–7.52), B = 9.68 Mya (95% HPD; 5.87–10.04), C = 10.51 Mya (95% HPD; 6.65–15.43). The split between the Hipposideridae (outgroup) and the Rhinolophidae together with fossil dates for *R. ferrumequinum* were used as calibration points; calibration points are indicated on the tree with an asterisk *. Sequences from the type localities for *R. darlingi darlingi* (TM2476) and *R. darlingi damarensis* (TM9474) are indicated on the tree in bold. The eastern *R. darlingi darlingi* subspecies forms a single clade together with *R. fumigatus*, *R. eloquens* and *R. hildebrandti* (blue) while *R. damarensis damarensis*, distributed in the western region of southern Africa, is composed of two very well supported clades that subdivide into northern and southern lineages (red) that diverged in the late Miocene.

**Figure 3:**
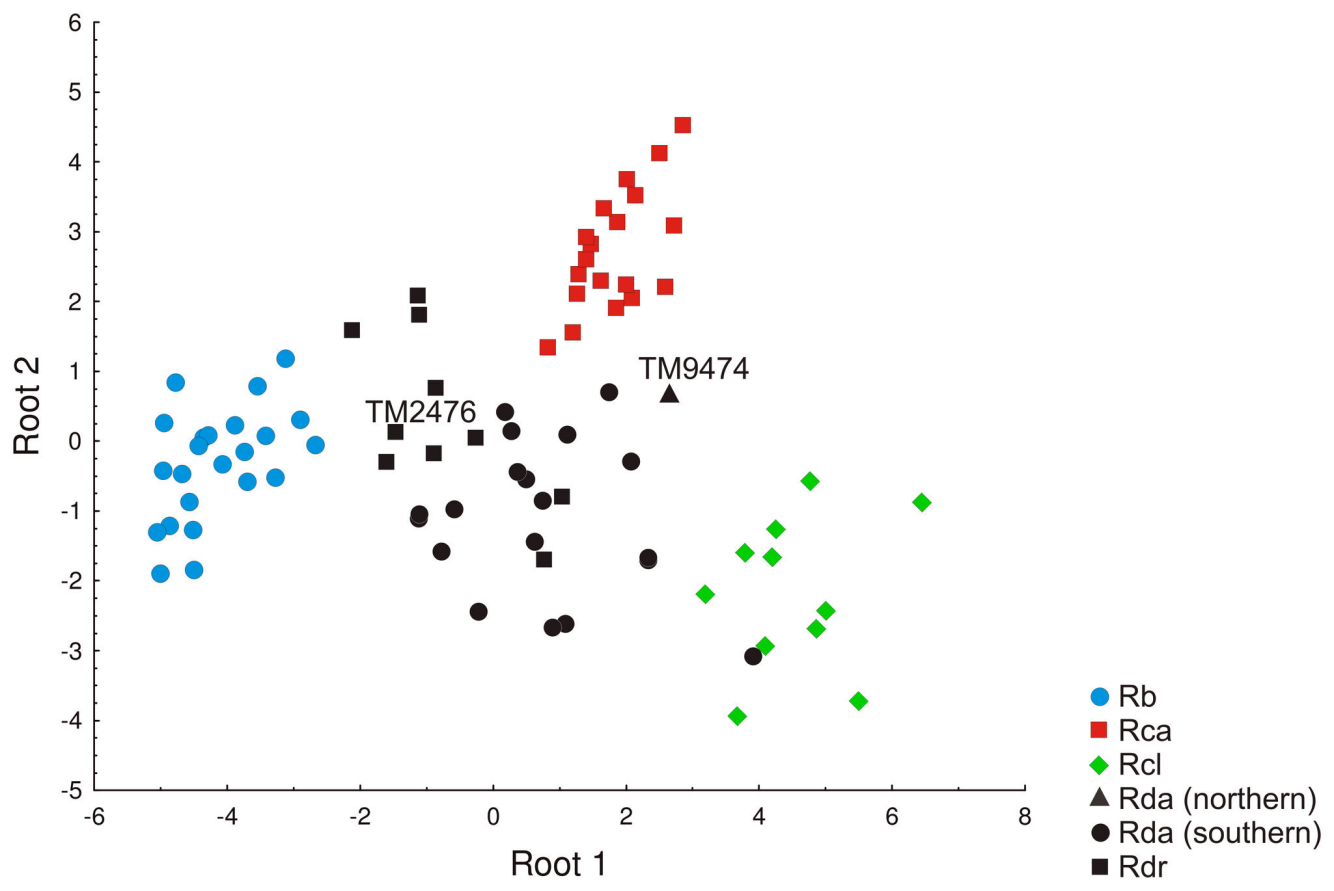
Plot of canonical scores extracted by Discriminant Function Analysis from 24 skull and external parameters. Species abbreviations are the same as those in Table S1 (Supporting Information). Museum accession numbers relate to holotypes (See legend of Figure 1).

Factorial analysis of variance (factorial ANOVA) was done on the echolocation data (Table S1 and Appendix S1 in File S1 Supporting Information) with resting frequency as the dependent variable and species (as identified in Figures 2 and 3) and sex as the categorical predictors. Noseleaf widths were also compared with a similar factorial ANOVA. All morphometric and echolocation analyses were done in Statistica v10 [57]. 

### Ecological niche modelling

Environmental variables ([58] - downloaded in October 2012 from The WorldBioclim website: http://www.worldclim.org/current) and locality records (this study; [42]) were geo-referenced (ArcGIS 10, ESRI) and used in the maximum entropy modelling technique (MaxEnt v3.3.3k; [59]). This was done to predict the environmental limits of the geographic ranges of the phylogenetic groups identified by the morphological and genetic analyses. The parameter settings we used are given in Supporting Information (Appendix S2, File S1).

## Results

### Phylogenetic reconstruction

In the cyt b sequences 42 % of the 951 cyt b sites were variable and 34% parsimony informative. 

The topology of the cyt b phylogenetic tree in Figure 2 was recovered repeatedly using all three methods of analyses (MP, ML and Bayesian inference). Discrepancies occurred only in the internal topology of the *Hipposideros* spp. outgroup in the ML and MP analyses in which *H. pratti* and *H. ruber* are sister taxa in a clade which is a sister clade to *H. bicolor*. Furthermore, the position of the Asian species *R. affinis* and *R. hipposideros* was unresolved in both ML and MP analyses but not in the Bayesian analysis. The MP analyses contained several polytomies so that the Asian clade could not be resolved from the African/European clade, the two European species, *R. euryale* and *R. mehelyi*, were not resolved within the African/European clade and the two African species, *R. eloquens* and *R. fumigatus* were not resolved within the *damarensis*/*fumigatus* clades. However, resolved nodes were generally well supported (high minimum bootstrap values of 70% in ML and 65% in MP analyses and posterior probabilities of 0.80–1.0 for the majority of nodes for the Bayesian inference) throughout the tree and we were able to date the major nodes of interest. The analysis revealed that *R. darlingi* (*sensu lato*) is a paraphyletic taxon; eastern and western populations of *R. darlingi* are placed in two non-sister clades which diverged ~9.68 Mya (Node B, Figure 2; 95% HPD: 5.87–10.04). The eastern population of *R. darlingi* is nested within the classic *fumigatus* clade which includes *R. fumigatus*, *R. hildebrandtii* and *R. eloquens*. Since the type locality of *R. darlingi* is in Zimbabwe we refer to this eastern population as *R. darlingi*. The second clade comprised individuals sampled in the western regions of South Africa and Namibia. This group of individuals included the holotype of the subspecies *R*. *d. damarensis* (TM 9474) collected from Oserikari, Namibia. We thus refer to this clade as the *damarensis* clade. The *damarensis* clade consists of two lineages, northern and south-central, which diverged ~4.8 Mya (Node A, Figure 2; 95% HPD: 2.69–7.52). The northern lineage is restricted to northern Namibia while the southern lineage includes individuals from central Namibia and north-western, south-western and central South Africa (Figure 2)

 The THY marker showed substantially less nucleotide variability. From 77 individuals 43 unique sequences were identified with 37% of the sites variable and 18% parsimony informative. All three phylogenetic inference methods failed to resolve clear relationships using the THY data set, even at higher taxonomic levels. The resulting trees had numerous polytomies and low bootstrap values (data not shown). The combined cyt b + THY analysis, using a subset of 13 *R. darlingi* individuals together with 15 additional rhinolophid species for which both markers were available, confirmed the relative topology of the two clades as recovered in the cyt b tree (Figure S1, Supporting Information).

The cyt b percentage sequence divergence (based on uncorrected p-distances) between the *darlingi* and *damarensis* clades was 8.1% and within each clade 1.3% and 2.1%, respectively. In comparison, the percentage sequence divergence between the *ferrumequinum* clade and the *darlingi* and *damarensis* clades was 9.6% and 7.9%, respectively. The northern and southern ‘*damarensis*’ lineages (Figures 1 and 2) had a percentage sequence divergence of 4.1% compared to 0.7% and 0.5%, respectively, within each of these lineages. 

### Morphometric analyses

#### Skull and post-cranial morphometrics

Despite the marked genetic divergence there was overlap in morphology between the *damarensis* clade and *R*. *d. darlingi* along both Roots 1 and 2 in the DFA (Figure 3). The first two roots extracted by DFA on the three principal components extracted by PCA (see Table S5 and Appendix S1 in File S1 Supporting Information) had Eigenvalues ≥ 1 and explained 98% of the variance. Classification success was > 80% for all groups. Principal component 1 (associated mainly with size) loaded highest on Root 1 and Principal component 2 (associated with palate length and inter-orbital width) loaded highest on Root 2 (Table 1). The *damarensis* and *R. darlingi* clades were separated by a Mahalanobis distance that was less than half that of the minimum distance between other groups (6.7 as opposed to 14.1, Table 2). 

**Table 1:** Results of discriminant functions analysis on principal component scores extracted by principal component analyses on 24 skull and external variables (see Table S5, Supporting Information).

	Root 1	Root 2	Root 3	Wilks' λ	F_(15, 210)_	p
PCA Factor 1	-0.84	0.42	0.34	0.2	126.71	< 0.0001
PCA Factor 2	-0.18	-0.98	0.02	0.09	49.69	< 0.0001
PCA Factor 3	-0.05	0.02	-1	0.03	6.04	< 0.0001
Eigenvalue	9.08	2.74	0.24			
Cumulative %	75.3	98	100			
Wilks' λ	0.02	0.22	0.81			
χ^2^	301.81	120.45	16.96			
df	15	8	3			
p	0.0001	0.0001	0.0001			

**Table 2:** Squared Mahalanobis distances obtained from Discriminant Function Analysis on skull and post-cranial measurements.

	*R. blasii*	*R. capensis*	*R. clviosus*	*R. d. damarensis*	*R. d. darlingi*
*R. blasii*		44.9	73.5	25.3	14.1
*R. capensis*	44.9		29.0	14.9	14.7
*R. clivosus*	73.5	29.0		12.8	32.7
*R. d. damarensis*	25.3	14.9	12.8		6.7
*R. d. darlingi*	14.1	14.7	32.7	6.7	

*1* Mahalanobis distances F’s_(3, 76)_ >8.4, p’s<0.0001

Several size-related variables, including forearm, skull length, mandible length and length of several digits were good discriminators between the other species (*R. clivosus*, *R. capensis* and *R. blasii*) as well as between these species and the *damarensis* and *darlingi* lineages (Root 1, Figure 3); the species with the largest dimensions for these variables (*R. clivosus*) was placed on the extreme right of Root 1 (Figure 3; Table S1 and Appendix S1 in File S1 Supporting Information) and the species with the smallest dimensions (*R. blasii*) on the extreme left of Root 1 (Figure 3; Table S1 and Appendix S1 in File S1 Supporting Information). However, there was not much difference in size between the *damarensis* and *R. darlingi* clades. Although *damarensis* was slightly bigger (e.g. in forearm and skull length) than *R. darlingi*, the ranges of their forearms and skull lengths overlapped (Table S1 and Appendix S1 in File S1 Supporting Information). Palate length and inter-orbital width (Table S1 and Appendix S1 in File S1 Supporting Information) were largely responsible for the separation of *R. capensis* from the other species along Root 2 (Figure 3). The *damarensis* clade included the holotype of the sub-species *R*. *d. damarensis* (TM 9474) and all the wild caught specimens from Namibia and central, north-western and south-western South Africa (Figures 1 and 2). The group labelled *R. darlingi* included all specimens from localities east of Taung (Figure 1), including the holotype of *R. d. barbetonensis* (TM 2476; Figure 1).

#### Noseleafs

The ranges of NLW of the *damarensis* clade and *R. darlingi* overlapped considerably (Table S1 and Appendix S1 in File S1 Supporting Information) despite significant differences amongst the species considered here (Table S1 and Appendix S1 in File S1 Supporting Information; factorial ANOVA F_(4,375)_ = 30.5, p<0.0001). Neither the difference between sexes nor the interactive term between sex and species was significant (factorial ANOVA F_(4,375)_<1.6, p>0.2) indicating that there were no differences in NLW between the sexes.

#### Baculum morphology

**Figure 4:**
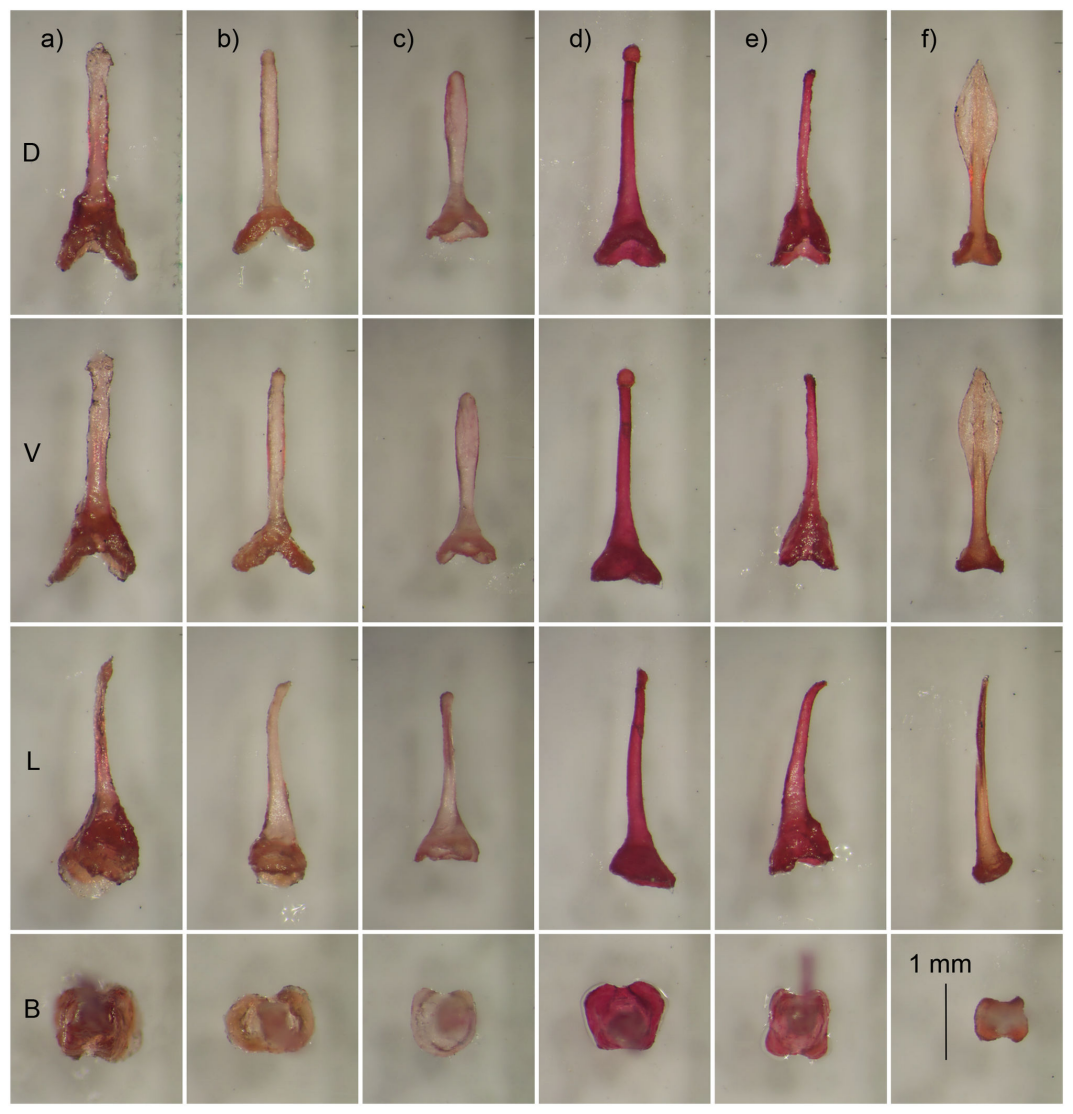
The bacula of five species of Rhinolophidae. Dorsal (row D), ventral (V) and lateral (L) views. Row B is the dorsal view of the base of each baculum. a) *R. damarensis* (Orange River), b) *R. damarensis* (Taung, TM 48040), c) *R. darlingi* (TM 47947), d) *R. capensis* (TM 40574), e) *R. blasii* (TM 7080), f) *R. clivosus* (TM46882). All figures are to the same scale and the scale line (bottom right) = 1 mm.

The shape and length of the bacula (Figure 4) largely supported the clades revealed in the phylogeny (Figure 2). The baculum of individuals from the *damarensis* clade (Figure 4a) had a short, dorso-ventrally flattened basal cone with dorsal and ventral incisions and the sides of the shaft tended to be more convex before tapering to a point (Figure 4c). The baculum of the *damarensis* specimen from the centre of South Africa (Taung, TM 48040; Figure 4b) was similar to those from north western South Africa (Vioolsdrif) but was slightly shorter and the shaft tip appears to be narrower. The cone formed by the base was also more flattened. 

There were significant difference in bacula measurements between the species (Figure 4c; Table S1 and Appendix S1 in File S1 Supporting Information; MANOVA F _(35.6, 36)_ = 8.38, p < 0.001) and the bacula of *R. darlingi* were shorter (mean = 2.58 mm) than that of *damarensis* (mean = 3.33 mm; post-hoc Tukey HSD tests p = 0.01; see Table S6 and Appendix S1 in File S1 Supporting Information). In addition, the dorsal view of the base was narrower (mean = 0.86 mm) than that of *damarensis* (mean = 1.29 mm; p = 0.02; see Table S6 and Appendix S1 in File S1, Supporting Information), and the dorsal incision on the base was shorter (mean = 0.33 mm) than that of *damarensis* (mean = 0.70 mm; p = 0.006; see Table S6 and Appendix S1 in File S1 Supporting Information). All other dimensions were not significantly different (all p > 0.2) between these two groups.

The bacula of the remaining species viz. *R. capensis*, *R. blasii* and *R. clivosus* (Figures 4d, 4e and 4f, respectively) were markedly different from each other and from the bacula of the *damarensis* clade and *R. darlingi*. 

### Echolocation frequency

The resting frequency differed amongst all species (factorial ANOVA F_(9, 379 )_ = 166.0; p<0.0001; Tukey test, all p<0.001) with the exception of the resting frequencies (Table S1 and Appendix S1 in File S1 Supporting Information) between the *damarensis* clade and *R. darlingi* (Tukey test, p>0.05) and between *R. blasii* and *R. clivosus* (Tukey test p>0.7). There was considerable overlap in the resting frequencies of *damarensis* and *R. darlingi* (Table S1 and Appendix S1 in File S1 Supporting Information). Resting frequency also did not differ significantly between sexes (F_(1, 379)_ = 3.0; p>0.05; Table S1 and Appendix S1 in File S1 Supporting Information). The interaction between species and sex was also not significant (F_(4, 379)_ = 1.0; p>0.05). 

### Ecological niche modelling

**Figure 5:**
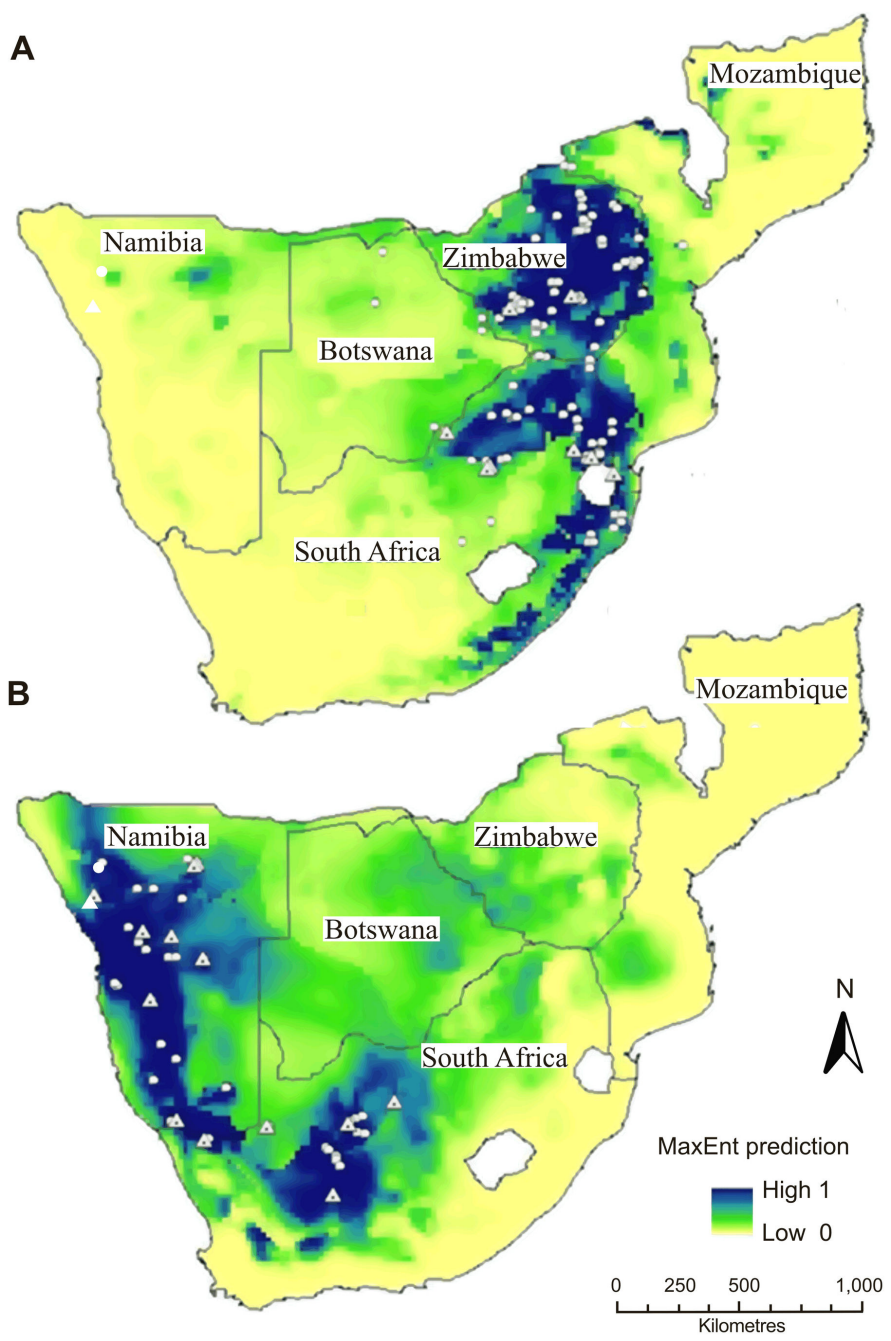
Probability of occurrence for A) *R. darlingi* and B) *R. damarensis* in southern Africa. The predictions (Low-High) show a suitability gradient from 0–1 based on environmental suitability where 0 (low) indicates a zero probability of occurrence and 1 (high) indicates maximum probability of occurrence. White circles indicate locality records used for modelling the species. Dotted white triangles indicate locality records from where DNA sequences were obtained. Variables used in the final model include annual mean temperature, isothermality, temperature seasonality, temperature annual range, mean temperature of the wettest quarter, mean temperature of the driest quarter, mean temperature of the warmest quarter, mean temperature of the coldest quarter, annual precipitation, precipitation seasonality, precipitation of the wettest quarter, precipitation of the driest quarter, and precipitation of the coldest quarter.

Areas with a high probability of occurrence for *R. darlingi* covered the eastern part of the southern African sub-region, mostly Zimbabwe and the north-east and eastern parts of South Africa (Figure 5a). In contrast, areas of high probability of occurrence for the *damarensis* clade covered the western part of the sub-region including Namibia and the driest parts of north-western, south-western and central South Africa (Figure 5b).

**Figure 6:**
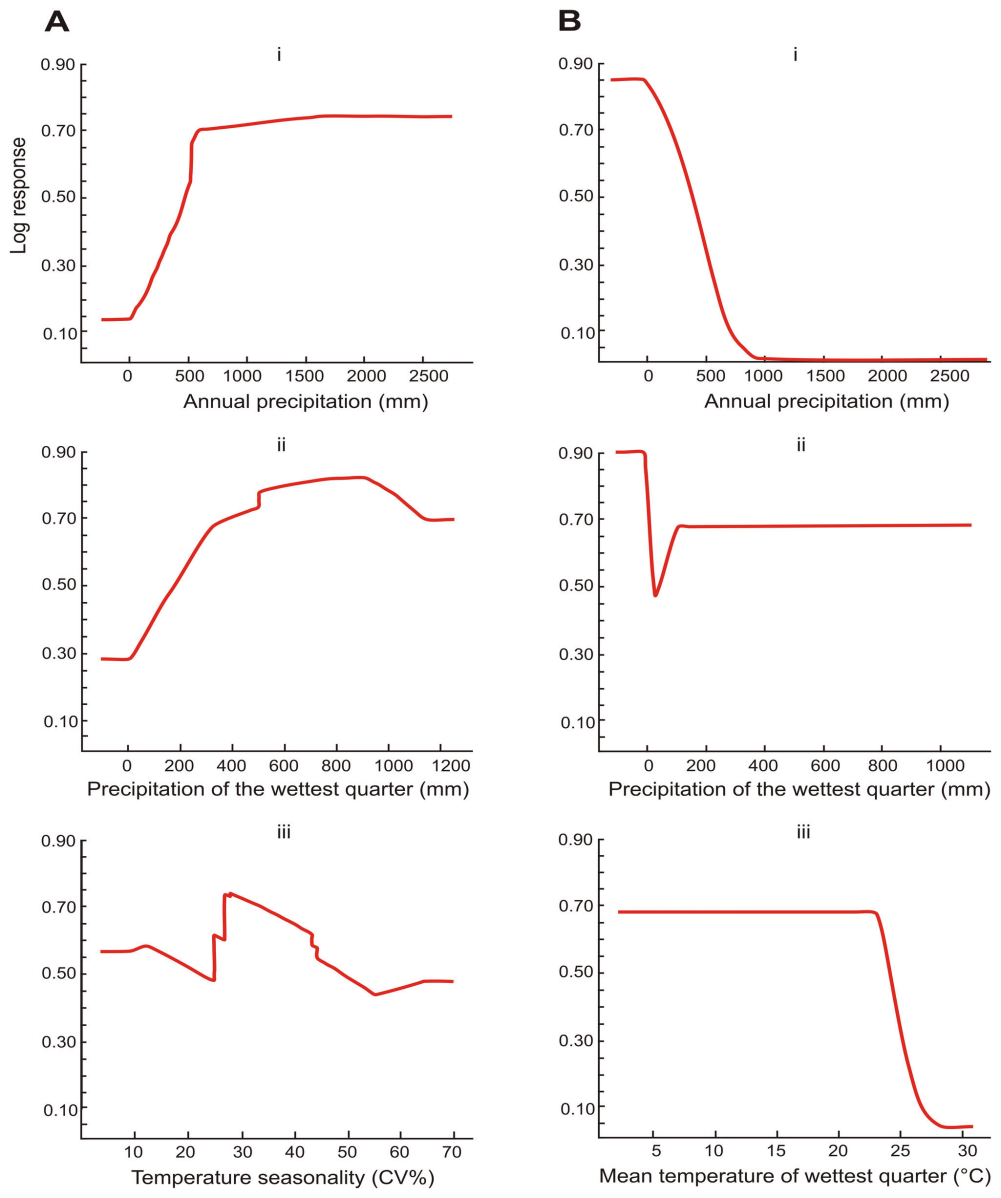
The log response curves for *R. darlingi*. A) *R. darlingi* ; (i) annual precipitation, (ii) precipitation of the wettest quarter, (iii) temperature seasonality when all locality records were used to build the model (temperature seasonality (Coefficient of Variation) is the standard deviation of the monthly temperature estimates expressed as a percentage of the mean of those estimates (i.e., the annual mean); and B) *R. damarensis*; (i) annual precipitation, (ii), precipitation of the wettest quarter, and (iii) mean temperature of the wettest quarter when all locality records were used to build the model.

The distribution of *R. darlingi* (Figure 5a) appeared to be limited by an annual precipitation of < 500mm (Figure 6a i) and favoured by precipitation in the wettest quarter ranging between 500–1100mm (Figure 6a ii). In addition, the distribution of this species was also limited by temperature seasonality of <10 and >55% and favoured by temperature seasonality of about 25 to 35% (Figure 6a iii).

The probability of occurrence of the *damarensis* clade reached zero when annual precipitation was >1000mm (Figures 5b and 6b i). In addition, the distribution of this species was also limited by a precipitation of the wettest quarter > 100 mm and favoured by a precipitation of about 0 mm in the wettest quarter of the year (Figure 6b ii). Furthermore, the probability response for the *damarensis* clade declined markedly to its lowest level of about < 0.05 when the mean temperature of the wettest quarter was about 28°C (Figure 6b iii). 

## Discussion

We explored the evolutionary history and ecology of *R. darlingi* (*sensu lato*) in the context of phenotypic convergence across its distributional range in southern Africa. Phylogenetic inference indicates that *R. darlingi* is in fact polyphyletic, comprising two non-sister lineages with disjunct distributions in southern Africa (Figures 1 and 5). Despite the genetic divergence between these two lineages echolocation frequencies as well as skull and post-cranial morphology, with the exception of the bacula, are highly convergent. 

### Systematics of *R. darlingi*

Eastern *R. darlingi*, which includes the holotype of *R. d. barbetonensis* (TM 2476), are genetically distinct from western *R. darlingi*, the two groups occurring in different clades. Eastern *R. darlingi* is embedded in the *fumigatus* clade that includes *R. fumigatus*, *R. eloquens* and *R. hildebrandtii*. Western *R. darlingi* form a monophyletic clade which diverged from the *fumigatus* clade ~9.7 Mya (Figure 2). Furthermore, the percentage sequence divergence for cyt b within eastern *R. darlingi* (1.3%) and within western *R. darlingi* (2.1%) was much lower than between the two clades (8.1%). This percentage sequence divergence falls within the range used to distinguish other species of bats (3–27%; [20,45]). This divergence was supported by differences in the size and shape of the bacula, characteristics which may act as crucial pre-zygotic reproductive barriers [60]. 

Ecological niche modelling showed substantial differences in the ecology of the two lineages. The distribution of the western lineage (*damarensis*; Figure 5) is restricted to the drier, hotter western half of the subcontinent (Figures 5b and 6b) characterised by an annual precipitation of < 500 mm and a mean temperature in the wettest quarter of about 28°C (Figure 6b). The distribution of eastern lineage (*R. darlingi*; Figure 5a) is restricted to the more mesic eastern half of the subcontinent (Figures 5a and 6a) suggesting a limited tolerance to arid conditions and a general absence from regions with an annual precipitation of < 100 mm and a temperature seasonality < 10% and > 55% (Figure 6a). 

Thus, there are species level genetic differences supported by differences in bacula morphology as well as substantial ecological differences between *R. darlingi* in the east and *R. darlingi* in the west, suggesting that a taxonomic revision is required despite their substantial convergence in echolocation frequency and skull and post-cranial morphology. Since the type specimen of *R. darlingi* was collected in Zimbabwe (eastern part of the subcontinent) we retain the name *R. darlingi* for the eastern lineage (Figure 1). Henceforth we refer to individuals in the western lineage as *R. damarensis* because the holotype of *R*. *d. damarensis* (TM9474, locality Namibia; Table S2 and Appendix S1 in File S1 Supporting Information) is associated with this group (Figure 3). The distribution of *R. darlingi* is restricted to the mesic eastern parts of the sub-continent (Figure 5a) and is described in detail in Monadjem et al. [42]. It appears to occupy mainly woodland and grassland biomes (Figure 5a; [41,42]). The distribution of *R. damarensis* is restricted to the xeric regions from south-western Angola, through northern Namibia, southwards as far as Carnarvon in south-western South Africa and occupies mainly arid savanna, Succulent- and Nama-Karoo, shrubland and desert (Figure 5b; [41,42]). The eastern limits of the distribution of *R. damarensis* appears to be demarcated by the area around Taung, the eastern most locality for a specimen in *R. damarensis* (TM48040, Table S2 and Appendix S1 in File S1 Supporting Information; Figure 1) and close to the boundary between the Savanna biome in the west and the Grassland biome in the east [41]. 

 The *R. damarensis* clade comprises two lineages, a northern lineage restricted to the more mesic regions of northern Namibia and a southern lineage with a distribution across several biomes in central and north-western South Africa, extending as far north as central Namibia (Figure 1). The genetic differentiation between the two lineages in *R. damarensis* is of the same magnitude as that used to infer cryptic species in other rhinolophids e.g. *R. arcuatus* [61]. In combination with ecological divergence the genetic divergence reported here suggests that *R. damarensis* may itself consist of cryptic species and further taxonomic revision of both this clade and *R. darlingi* (*sensu lato*) is required.

### Morphological and acoustic convergence

Despite the relatively ancient split (~9.7 Mya), marked genetic differentiation and the occupation of different biomes there was convergence in the cranial and post-cranial measurements of *R. damarensis* and *R. darlingi* (Figure 3, Table S1 and Appendix S1 in File S1 Supporting Information) as well as in the noseleaf width and resting echolocation frequency (Table S1 and Appendix S1 in File S1 Supporting Information). None of these parameters are thus taxonomically informative with respect to differentiating *R. damarensis* from *R. darlingi*. The phenotypic similarity between the two *darlingi* lineages is greater than that between any other pair of species in our analyses (Table 2). Furthermore, *R. darlingi* is more similar to *R. damarensis* in body size and echolocation frequency than it is to any of the other species in the *fumigatus* clade (Figure 2) which all have bigger body sizes and lower resting echolocation frequencies (than both *R. darlingi* and *R. damarensis*) ranging from 48–67 mm (forearm length) and 32–54 kHz, respectively [31,42]. They are also more similar to each other than either is to the ancestral character state at node B (Figure 2; forearm length = 58.7 mm; resting frequency = 60.1 kHz [39]). 

Such convergence involving morphology and echolocation in non-sibling species deviates from the pattern normally found in cryptic species of bats in general and rhinolophids in particular. All cryptic bat species uncovered so far have similar morphology but divergent echolocation frequencies which differed by up to 13 kHz (e.g. [19,20,62], however see 61). These cryptic species all co-occurred and such differences in sensory traits may be important isolating mechanisms between species [63] leading to resource partitioning and subsequent genetic divergence. At lower echolocation frequencies, where differences in frequency translate into large differences in wavelength [64], habitat and insect prey may be partitioned [27,36,65,66]. At higher frequencies, where differences are unlikely to equate to marked differences in wavelength, resource partitioning may be mediated by the selection for discrete frequency bands to facilitate intraspecific communication [29,33,34,64]. The call frequency of one or more of the co-existing species may shift so that individuals are more sensitive, and will respond preferentially, to the calls of their own species [27,29], facilitating intraspecific communication. Divergence in echolocation calls in sympatry may therefore be a consequence of competition leading to character displacement in at least one phenotypic trait that permits resource partitioning and coexistence. There is at least one example where sympatric bat lineages converge in both morphology and echolocation [61], attributed to either novel niche partitioning or recent contact. Here lineages within the rhinolophid *R. arcuatus* may have partitioned their niches in novel ways along dimensions not previously considered. Alternatively, convergence may have evolved in allopatry with the two lineages recently making contact [61]. 

Strong convergence in allopatry may be a consequence of lineages evolving in the absence of competition from ecologically similar species; their phenotypes being the result of neutral evolution or shaped by selection pressures resulting from occupying similar niches albeit in different biomes. The disjunct distribution of *R. damarensis* and *R. darlingi* may allow their morphology and echolocation to converge because they do not compete for foraging space, prey or discrete frequency bands. Such convergence may result from one or more of several processes including inheritance from a common ancestor, adaptation to similar local environments, random genetic drift and shared constraints [5,67]. Inheritance from a recent common ancestor is unlikely to explain the phenotypic convergence between *R. darlingi* and *R. damarensis*. They are placed in different, albeit sister clades: *R. damarensis* in its own clade and *R. darlingi* in the *fumigatus* clade (Figure 2). The two lineages last shared a common ancestor ~9.7Mya, giving rise to numerous lineages comprising individuals that are bigger in size and echolocate at lower frequencies than either *R. darlingi* and *R. damarensis* viz. *R. fumigatus*, *R. eloquens* and *R. hildebrandtii* [42]. Similarly, the fact that there are species that share a common ancestor with *R. damarensis* but that are nevertheless divergent in both morphology and echolocation appears to exclude constraints as an explanation for the convergence. Local adaptation also appears to be an unlikely explanation for the convergence because the two species occur in different biomes and it would be expected that local adaptation would lead to divergence not convergence. It is therefore likely that convergence may be the result of random genetic drift especially since rates of convergence can be high when lineages are diverging only under the influence of genetic drift [6]. Testing this hypothesis would require thorough and integrated analyses of genetic and phenotypic variation in both the *damarensis* and the *fumigatus* clades (Figure 2). Nevertheless, there is some evidence that founder effect and random genetic drift may be implicated in the evolution of different body sizes during the diversification of the *R. hildebrandtii* species-complex [31], one of the lineages in the *fumigatus* clade – this clade also includes *R. darlingi* (Figure 2). If so, smaller body size in *R. darlingi* may have evolved through genetic drift resulting in the convergence of body size between it and *R. damarensis*, assuming that the ancestral body size of *R. damarensis* is similar to its current body size. Similar body sizes, coupled with the unique flutter-detection system of rhinolophids [68], would require similar detection distances and levels of flight manoeuvrability that could lead to convergence in wing morphology and echolocation frequency and possibly also insect prey types. This may be especially so given the well-established correlations between body size on the one hand and wing loading, echolocation frequency and bite force, on the other, in bats [63,69–71]. Bite force is in turn correlated with diet [70]. 

The split between the two *damarensis* lineages provides further insight into the role of random genetic drift in the evolution of rhinolophids in southern Africa. The split occurred ~5 Mya (Figure 2) which is similar to divergence times reported in many co-distributed taxa including the African four-striped mouse (*Rhabdomys pumilio*, [72]), the southern rock Agama (*Agama atra*, [73]) and the gecko, *Pachydactylus rugosus* [74]. Similarity in the timing of evolutionary diversification amongst co-distributed but diverse taxa is likely a consequence of vicariant evolution [75] and in southern Africa this has been attributed to climate change and subsequent vegetation shifts during the Plio-Pleistocene and Miocene [72–74,76] together with the Plio-Pleistocene uplift of southern Africa’s great escarpment and interior plateau [77]. Diversification across these lineages coincided with a period of increased aridity in southern Africa as a result of the interaction in the Miocene between global cooling [78] and tectonic uplift that resulted in a topography which sloped from east to west causing a rain-shadow effect across the region [79,80]. This in turn resulted in an east-west gradient of rainfall and subsequent changes in vegetation which included the contraction of forests, and the expansion of savanna woodlands, grasslands and shrublands [81–83] towards the end of the Miocene (7–5Mya). Such climatic oscillations and habitat fluctuation/fragmentation promote diversification of lineages. The diversification of *R. damarensis* into two distinct mitochondrial lineages may have been caused by disruption to gene flow associated with these changes in biomes especially since the lineages currently occupy separate geographic regions. Given that rates of convergence can be high when lineages diverge under the influence of genetic drift [6] convergence in phenotype in the two *damarensis* lineages would not be surprising if drift was the dominant process acting during their initial divergence. Testing this hypothesis and the relative influence of the different processes that could bring about convergence can only be elucidated through thorough and integrated analyses of both genetic and phenotypic variation using multiple rapidly and slowly evolving genetic markers, within the context of historical biogeography.

In conclusion, cryptic lineages in *R. darlingi* (*sensu lato*) appear to have arisen independently and in isolation of each other allowing convergence in both morphology and echolocation. Similarly, cryptic lineage diversification within *R. damarensis* also appears to have arisen more recently in response to changes in biome boundaries during the Miocene. Although this might be due to vicariant evolution the role of other processes such as adaptation as a result of occupying similar niches cannot be excluded at this stage. 

## Supporting Information

File S1 **Supplementary tables, methods and results.**
(DOCX)

Figure S1 **Bayesian consensus topology of *Rhinolophus* spp. based on a combined analysis of cyt b and THY on a subset of species for which both markers were available.**
(TIF)
