# Supplementary material for: Phenotypic Convergence in Genetically Distinct Lineages of a Rhinolophus Species Complex (Mammalia, Chiroptera)
Source: PLoS One. 2013 Dec 3;8(12):e82614. doi: 10.1371/journal.pone.0082614 (PMC3849494; doi:10.1371/journal.pone.0082614)
Supplement: File S1 — Supplementary tables, methods and results. (DOCX) [file pone.0082614.s001.docx]

**SUPPORTING INFORMATION FILE S1**

**Appendix S1 Supplementary tables.**

**Table S1.** The means ± standard deviations, ranges and sample sizes of skull and external measurements (in mm) and resting echolocation frequency (RF; in kHz) for several species of southern African rhinolophids.

|  | ^1^*Rb* | *Rca* | *Rcl* | *Rda* | *Rdr* |
| --- | --- | --- | --- | --- | --- |
| ^2^ALSW | 5.1 ± 0.1 | 5.4 ± 0.2 | 5.7 ± 0.1 | 5.5 ± 0.2 | 5.2 ± 0.1 |
|  | 4.9-5.3 | 5.2-5.8 | 5.5-6.0 | 4.9-5.2 | 4.9-5.4 |
|  | 23 | 19 | 12 | 20 | 10 |
| AMSW | 3.3 ± 0.1 | 3.6 ± 0.2 | 3.5 ± 0.2 | 3.6±0.1 | 3.6 ± 0.2 |
|  | 3.2-3.4 | 3.1-3.8 | 3.3-3.8 | 3.3-3.9 | 3.3-3.9 |
|  | 23 | 19 | 12 | 20 | 10 |
| UCM3L | 6.7 ± 0.1 | 7.6 ± 0.1 | 8.0 ± 0.2 | 7.4 ± 0.1 | 7.1 ± 0.2 |
|  | 6.4-6.9 | 7.4-7.8 | 7.6-8.4 | 7.1-7.7 | 6.8-7.4 |
|  | 23 | 19 | 12 | 20 | 10 |
| IOW | 2.5 ± 0.1 | 2.4 ± 0.1 | 2.9 ± 0.2 | 2.7 ± 0.2 | 2.5 ± 0.1 |
|  | 2.4-2.7 | 2.2-2.6 | 2.6-3.3 | 2.3-3.1 | 2.3-2.7 |
|  | 23 | 19 | 12 | 20 | 10 |
| M3M3W | 6.3 ± 0.1 | 7.5 ± 0.2 | 8.3 ± 0.3 | 7.5±0.2 | 7.4 ± 0.1 |
|  | 6.1-6.5 | 7.3-8.0 | 7.6-8.8 | 7.2-7.9 | 7.2-7.6 |
|  | 23 | 19 | 12 | 20 | 10 |
| MW | 9.1 ± 0.2 | 10.0 ± 0.2 | 10.1 ± 0.2 | 9.6 ± 0.4 | 9.2 ± 0.2 |
|  | 8.7-9.5 | 9.7-10.4 | 9.9-10.4 | 9.2-10.9 | 8.9-9.4 |
|  | 23 | 19 | 12 | 20 | 10 |
| PL | 2.3 ± 0.1 | 2.5 ± 0.1 | 2.1 ± 0.1 | 2.3 ± 0.2 | 2.3 ± 0.2 |
|  | 2.1-2.7 | 2.3-2.6 | 2.0-2.3 | 2.0-2.6 | 2.0-2.6 |
|  | 23 | 19 | 12 | 20 | 10 |
| SL | 18.8 ± 0.3 | 20.6 ± 0.3 | 21.6 ± 0.5 | 20.2 ± 0.5 | 19.3 ± 0.4 |
|  | 18.3-19.2 | 20.1-21.2 | 20.6-22.4 | 19.5-21.1 | 18.6-19.6 |
|  | 23 | 12 | 20 | 10 |  |
| ZW | 8.9 ± 0.2 | 10.5 ± 0.2 | 11.4 ± 0.4 | 10.3 ± 0.3 | 10.1 ± 0.3 |
|  | 8.6-9.2 | 10.2-10.9 | 10.5-12.0 | 9.8-10.9 | 9.7-10.4 |
|  | 23 | 23 | 12 | 20 | 10 |
| ML | 11.9 ± 0.2 | 13.6 ± 0.3 | 14.6 ± 0.4 | 13.6±0.3 | 12.8 ± 0.3 |
|  | 11.6-12.2 | 13.0-14.1 | 13.9-15.5 | 13.0-14.1 | 12.4-13.2 |
|  | 23 | 19 | 12 | 20 | 10 |
| LCM3L | 6.9 ± 0.1 | 8.1 ± 0.2 | 8.7 ± 0.3 | 8.0 ± 0.2 | 7.5 ± 0.2 |
|  | 6.7-7.1 | 7.9-8.8 | 8.0-9.0 | 7.7-8.3 | 7.3-7.8 |
|  | 23 | 19 | 12 | 20 | 10 |
| FA | 44.9 ± 1.3 | 48.6 ± 0.8 | 53.1 ± 2.2 | 49.5 ± 1.7 | 47.2 ± 2.6 |
|  | 41.6-47.5 | 46.7-49.9 | 48.6-57.0 | 47.0-52.7 | 41.6-50.3 |
|  | 21 | 19 | 11 | 20 | 10 |
| M-III | 29.8 ± 1.0 | 33.1 ± 0.6 | 35.3 ± 1.7 | 32.1±1.1 | 30.5 ± 1.6 |
|  | 27.2-31.0 | 32.3-34.2 | 31.3-37.0 | 30.0-34.4 | 28.8-33.4 |
|  | 23 | 19 | 12 | 20 | 10 |
| P1-III | 14.4 ± 0.4 | 15.2 ± 0.6 | 17.5 ± 0.8 | 16.6 ± 0.6 | 15.3 ± 1.1 |
|  | 13.8-15.3 | 14.3-16.3 | 15.8-19.0 | 15.2-18.0 | 13.5-16.8 |
|  | 23 | 19 | 12 | 20 | 10 |
| P2-III | 23.8 ± 1.0 | 25.1 ± 0.9 | 28.7 ± 1.9 | 26.2 ± 1.3 | 24.6 ± 1.7 |
|  | 20.9-24.9 | 22.6-26.6 | 24.7-30.2 | 24.0-29.4 | 21.9-28.0 |
|  | 23 | 19 | 12 | 20 | 10 |
| M-IV | 32.2 ± 1.2 | 35.6 ± 0.7 | 38.7 ± 1.8 | 35.7 ± 1.4 | 33.7 ± 1.6 |
|  | 29.0-34.0 | 34.5-36.7 | 35.2-41.0 | 33.5-39.8 | 31.0-36.0 |
|  | 23 | 19 | 12 | 20 | 10 |
| P1-IV | 8.0 ± 0.3 | 8.5 ± 0.3 | 10.5 ± 0.7 | 9.4 ± 0.6 | 8.4 ± 0.6 |
|  | 7.6-8.6 | 7.9-9.2 | 9.1-11.7 | 8.5-10.8 | 7.6-9.6 |
|  | 22 | 19 | 12 | 20 | 10 |
| P2-IV | 14.5 ± 0.9 | 16.0 ± 0.5 | 18.1 ± 0.8 | 17.0 ± 1.2 | 15.5 ± 0.7 |
|  | 11.6-16.0 | 15.2-16.7 | 16.6-19.5 | 15.5-19.8 | 14.5-16.4 |
|  | 22 | 10 | 12 | 20 | 10 |
| M-V | 33.7 ± 1.0 | 35.7 ± 0.8 | 39.5 ± 1.6 | 36.4 ± 1.4 | 34.4 ±1.4 |
|  | 30.7-34.9 | 34.4-37.6 | 36.1-41.7 | 34.0-39.8 | 31.9-36.9 |
|  | 23 | 19 | 12 | 19 | 10 |
| P1-V | 10.6 ± 0.4 | 10.9 ± 0.4 | 12.8 ± 1.0 | 11.4 ± 0.7 | 10.3 ± 0.8 |
|  | 9.9-11.2 | 10.0-11.9 | 11.0-14.7 | 10.4-13.2 | 9.0-11.8 |
|  | 23 | 17 | 12 | 19 | 10 |
| P2-V | 11.8 ± 0.6 | 12.7 ± 0.8 | 15.3 ± 0.4 | 15.0 ± 1.0 | 14.2 ± 0.9 |
|  | 10.6-12.6 | 10.8-13.7 | 14.2-16.0 | 13.0-16.9 | 12.9-15.3 |
|  | 23 | 19 | 12 | 19 | 10 |
| TB | 17.7 ± 0.5 | 18.8 ± 0.9 | 21.6 ± 1.0 | 20.6 ± 1.0 | 19.3 ± 1.1 |
|  | 16.6-18.9 | 17.5-21.2 | 20.3-23.4 | 19.1-22.6 | 17.5-21.4 |
|  | 22 | 19 | 12 | 20 | 10 |
| TL | 24.4 ± 2.7 | 26.9 ± 1.6 | 31.3 ± 2.9 | 27.4 ± 3.6 | 29.5 ± 2.2 |
|  | 21.0-30.0 | 25.0-29.0 | 26.0-35.0 | 21.4-34.7 | 26.0-33.0 |
|  | 22 | 19 | 10 | 18 | 10 |
| EL | 17.7 ± 1.8 | 23.9 ± 1.3 | 20.9 ± 1.6 | 19.6 ± 1.7 | 19.0 ± 1.9 |
|  | 15.0-20.0 | 21.0-25.0 | 18.0-23.0 | 15.2-22.3 | 16.0-23.0 |
|  | 22 | 19 | 10 | 18 | 10 |
| NLW | 8.3 ± 0.7 | 8.5 ± 0.7 | 7.6 ± 0.5 | 8.8 ± 0.7 | 8.1 ± 0.5 |
|  | 7.0-9.4 | 7.1-11.0 | 6.3-8.5 | 7.0-11.0 | 7.4-9.1 |
|  | 32 | 185 | 47 | 106 | 15 |
| RF | 91.3 ± 0.9 | 82.8 ± 3.1 | 92.1 ± 0.8 | 85.4 ± 1.4 | 85.5 ± 2.7 |
|  | 89.0-93.0 | 73.6-87.1 | 90.0-93.3 | 82.2-89.1 | 79.3-88.6 |
|  | 32 | 185 | 47 | 110 | 15 |

^1^ Rb = *R. blasii*; Rca = *R. capensis*; Rcl = *R. clivosus*; Rda = *R. darlingi damarensis*; Rdr = *R. d. darlingi*.

^2^ Measurements are abbreviated as: Forearm length (FA); tail length (TL); tibia length (TB); ear length (EL); skull length (SL); mandible (ML); palate length (PL); the greatest width of the anterior lateral swellings in dorsal view (ALSW); width of the anterior median swellings in dorsal view (AMSW), zygomatic width (ZW); length of the upper tooth row including the upper canine and third molar (UCM3L); length of lower tooth row including the lower canine and lower third molar (LCM3L) inter orbital width (IOW); width of upper jaw across the two third molars (M3M3); mastoid width (MW); length of metacarpal of 3^rd^ digit (M-III); length of 1^st^ phalanx of 3^rd^ digit ((P1-III); length of 2^nd^ phalanx of 3^rd^ digit (P2-III); length of metacarpal of 4^th^ digit (M-IV); length of 1^st^ phalanx of 4^th^ digit (P1-IV); length of 2^nd^ phalanx of 4^th^ digit (P2-IV); length of metacarpal of 5^th^ digit (M-V); length of 1^st^ phalanx of 5^th^ digit (P1-V); length of 2^nd^ phalanx of 5^th^ digit (P2-V). The above measurements are illustrated in Csorba et al. (2003).

**Table S2.** Museum specimens of the genus *Rhinolophus* (*R*.) which were measured for this study.

| **Museum No** | **Species** | **Locality** | **GPS Co-ordinates** |  |  |
| --- | --- | --- | --- | --- | --- |
| TM 13962 | *R. blasii* | Sandspruit Cave, Limpopo | 24°37’04.9’’S | 27°39’07.9’E |  |
| TM 13963 | *R. blasii* | Sandspruit Cave, Limpopo | 24°37’04.9’’S | 27°39’07.9’’E |  |
| TM 13996 | *R. blasii* | Sandspruit Cave, Limpopo | 24°37’04.9’’S | 27°39’07.9’’E |  |
| TM 13997 | *R. blasii* | Sandspruit Cave, Limpopo | 24°37’04.9’’S | 27°39’07.9’’E |  |
| TM 13998 | *R. blasii* | Sandspruit Cave, Limpopo | 24°37’04.9’’S | 27°39’07.9’’E |  |
| TM 2887 | *R. blasii* | Farm Uitkomst 499 JQ, Gauteng | 25°55’S | 27°45’E |  |
| TM 2888 | *R. blasii* | Farm Uitkomst 499 JQ, Gauteng | 25°55’S | 27°45’E |  |
| TM 47084 | *R. blasii* | Farm Victoriaspoort 18 IT, MP | 26°07’S | 30°22’E |  |
| TM 47731 | *R. blasii* | Farm In-de-diepte 164 JT, MP | 25°43’00’’S | 30°01’00’’E |  |
| TM 47766 | *R. blasii* | Sandspruit Cave, Limpopo | 24°37’04.9’’S | 27°39’07.9’’E |  |
| TM 7063 | *R. blasii* | Uitkyk, Gauteng | 26°06’S | 27°46’E |  |
| TM 7066 | *R. blasii* | Uitkyk, Gauteng | 26°06’S | 27°46’E |  |
| TM 13960 | *R. blasii* | Sandspruit Cave, Limpopo | 24°37’04.9’’S | 27°39’07.9’’E |  |
| TM 13961 | *R. blasii* | Sandspruit Cave, Limpopo | 24°37’04.9’’S | 27°39’07.9’’E |  |
| TM 13995 | *R. blasii* | Sandspruit Cave, Limpopo | 24°37’04.9’’S | 27°39’07.9’’E |  |
| TM 13999 | *R. blasii* | Sandspruit Cave, Limpopo | 24°37’04.9’’S | 27°39’07.9’’E |  |
| TM 4263 | *R. blasii* | Makapansgat, Limpopo | 24°09’S | 29°11’E |  |
| TM 4265 | *R. blasii* | Makapansgat, Limpopo | 24°09’S | 29°11’E |  |
| TM 46645 | *R. blasii* | Sudwala Cave, MP | 25°22’S | 30°42’E |  |
| TM 47083 | *R. blasii* | Farm Victoriaspoort 18IT, MP | 26°07’S | 30°22’E |  |
| TM 7062 | *R. blasii* | Uitkyk, Gauteng | 26°06’S | 27°46’E |  |
| TM 7079 | *R. blasii* | Uitkyk, Gauteng | 26°06’S | 27°46’E |  |
| TM 7080 | *R. blasii* | Uitkyk, Gauteng | 26°06’S | 27°46’E |  |
| TM 29067 | *R. capensis* | De Hoop NR, WC | 34°26’S | 20°25’E |  |
| TM 29077 | *R. capensis* | De Hoop NR, WC | 34°26’S | 20°25’E |  |
| TM 29080 | *R. capensis* | De Hoop NR, WC | 34°26’S | 20°25’E |  |
| TM 40567 | *R. capensis* | De Hoop NR, WC | 34°26’S | 20°25’E |  |
| TM 8983 | *R. capensis* | Stilbaai, WC | 34°22’00’’S | 21°25’00’’E |  |
| TM 2275 | *R. capensis* | Klaver, WC | 31°46’59’’S | 18°40’36’’E |  |
| TM 40568 | *R. capensis* | De Hoop NR, WC | 34°26’S | 20°25’E |  |
| TM 29065 | *R. capensis* | De Hoop NR, WC | 34°26’S | 20°25’E |  |
| TM 29070 | *R. capensis* | De Hoop NR, WC | 34°26’S | 20°25’E |  |
| TM 29079 | *R. capensis* | De Hoop NR, WC | 34°26’S | 20°25’E |  |
| TM 40566 | *R. capensis* | De Hoop NR, WC | 34°26’S | 20°25’E |  |
| TM 40569 | *R. capensis* | De Hoop NR, WC | 34°26’S | 20°25’E |  |
| TM 40570 | *R. capensis* | De Hoop NR, WC | 34°26’S | 20°25’E |  |
| TM 40571 | *R. capensis* | De Hoop NR, WC | 34°26’S | 20°25’E |  |
| TM 40573 | *R. capensis* | De Hoop NR, WC | 34°26’S | 20°25’E |  |
| TM 40574 | *R. capensis* | De Hoop NR, WC | 34°26’S | 20°25’E |  |
| TM 40575 | *R. capensis* | De Hoop NR, WC | 34°26’S | 20°25’E |  |
| TM 40576 | *R. capensis* | De Hoop NR, WC | 34°26’S | 20°25’E |  |
| TM 41560 | *R. capensis* | Postberg NR, WC | 33°07’S | 18°00’E |  |
| TM 48347 | *R. clivosus* | 3 km NNE of Venterskroon, NW | 26°51’45.2’’ S | 27°17’00.7’’ E |  |
| TM48346 | *R. clivosus* | 3 km NNE of Venterskroon, NW | 26°51’45.2’’ S | 27°17’00.7’’E |  |
| TM 48344 | *R. clivosus* | 1.8 km NW of Venterskroon, NW | 26°52’39.3’’ S | 27°15’15.2’’E |  |
| TM 48345 | *R. clivosus* | 1.8 km NW of Venterskroon, NW | 26°52’39.3’’ S | 27°15’15.2’’E |  |
| TM 29062 | *R. clivosus* | De Hoop NR, WC | 34°26’S | 20°25’E |  |
| TM 46882 | *R. clivosus* | Farm Haffenden Heights 35KT, MP | 24°07’S | 30°07’E |  |
| TM 47613 | *R. clivosus* | Farm Sudwalaskraal 271JT, MP | 25°22’37.3’’S | 30°41’30.1’’E |  |
| TM 47653 | *R. clivosus* | Farm Berlin 446JT, MP | 25°30’47.6’’S | 30°46’28.6’’E |  |
| TM 47767 | *R. clivosus* | Bolts Farm, Gauteng | 26°02’05.2’’S | 27°42’48.2’’E |  |
| TM 47768 | *R. clivosus* | Farm Tweefontein 523 JQ, Gauteng | 25°56’12.1’’S | 27°49’28.5’’E |  |
| TM 48192 | *R. clivosus* | Schaapsplaats, FS | 28°34’28.4’’S | 28°27’09.4’’E |  |
| TM 47655 | *R. clivosus* | VerlorenValei NR, MP | 25°16’39.5’’S | 30°05’56.3’’E |  |
| TM 9474* | *R. d. damarensis* | Oserikari, Namibia | 21°56’S | 17°05’E |  |
| TM 48040* | *R. darlingi* | Taung, NW | 27°36’50.6’’S | 24°37’45.8’’E |  |
| TM 2476* | *R. d. barbetonensis* | Louw's Creek, Barberton, MP | 25°38’S | 31°18’E |  |
| TM 47095 | *R. darlingi* | Waterberg GR, Limpopo | 24°06’59.4’’S | 28°23’17.8’’E |  |
| TM 47661 | *R. darlingi* | Barberton, MP | 25°43’’09.2’S | 31°15’56.5’’E |  |
| TM 47757 | *R. darlingi* | Farm Tweefontein 523 JQ, Gauteng | 25°54’27.7’’S | 27°46’21.0’’E |  |
| TM 48018* | *R. darlingi* | Madikwe GR, NW | 24°45’58.3’’S | 26°22’15.5’’E |  |
| TM 48019* | *R. darlingi (J)* | Madikwe GR, NW | 24°45’58.3’’S | 26°22’15.5’’E |  |
| TM 48020* | *R. darlingi* | Madikwe GR, NW | 24°45’58.3’’S | 26°22’15.5’’E |  |
| TM 48021* | *R. darlingi (J)* | Madikwe GR, NW | 24°45’58.3’’S | 26°22’15.5’’E |  |
| TM 47765* | *R. darlingi* | Farm Tweefontein 523 JQ, Gauteng | 25°56’12.1’’S | 27°49’28.6’’E |  |
| TM 47742* | *R. darlingi* | Mlawula NR, Swaziland | 26°10’48.6’’S | 32°02’57.5’’E |  |
| TM 47744 | *R. darlingi* | Mlawula NR, Swaziland | 26°10’48.6’’S | 32°02’57.5’’E |  |
| TM 47947* | *R. darlingi* | Farm Sterkfontein 173 IQ, Gauteng | 26°01’43.4’’S | 27°42’37.2’’E |  |

FS = Free State Province, MP = Mpumalanga Province, NW = North West Province, WC = Western Cape Province, GR = Game Reserve, NR = Nature Reserve, J = juvenile, * = sequenced.

**Table S3.** List of specimens examined and sequences analysed in this study.

| Taxon | Locality | Tissue ID | Source | GenBank N^o^ | Gene (s) |
| --- | --- | --- | --- | --- | --- |
| *H. bicolour* | Thailand | AGS970408-03 | Guillen-Servent  & Francis 2006 | DQ054808 | Cytochrome b |
| *H. caffer* | South Africa | 17.12.02HC2(SDC) | Stoffberg *et al.* 2010 | FJ171187 | THY |
| *H. commersoni* | Zambia | DSJZM26 | Eick *et al.* 2005 | AJ865642 | THY |
| *H. pratti* | Presumably China |  | Niu *et al.* 2007 | EF544427 | Cytochrome b |
| *H. ruber* | Tanzania | WTS2030 | Stoffberg *et al.* 2010 | FJ185183, FJ171188 | Cytochrome b, THY |
| *R. acuminatus* | Myanmar | TM4 | Stoffberg *et al.* 2010 | FJ171189 | THY |
| *R. affinis* | China | 59477 | Sun *et al.* 2009 | EU434934 | Cytochrome b |
| *R. affinis* | Thailand | 99.512 | Stoffberg *et al.* 2010 | FJ171190 | THY |
| *R. affinis* | Myanmar | IL6 | Stoffberg *et al.* 2010 | FJ171191 | THY |
| *R. alcyone* | Ivory Coast | ROM-100491 | Zhou *et al.* 2009 | EU436667 | Cytochrome b |
| *R. blasii* | Morocco | EBD-23260 | Zhou *et al.* 2009 | EU436669 | Cytochrome b |
| *R. blasii* | Zimbabwe | 10062012RSi03MUS | This study | KF683216 | Cytochrome b |
| *R. blasii* | South Africa | 18.12.02RB2(SC) | Stoffberg *et al.* 2010 | FJ171192 | THY |
| *R. blasii* | Greece | MHNG1807.41 | Stoffberg *et al.* 2010 | FJ171193 | THY |
| *R. borneensis* | Cambodia | CSOCA17 | Stoffberg *et al.* 2010 | FJ171194 | THY |
| *R. capensis* | South Africa | DSJ5 | Eick *et al.* 2005 | AJ865672 | THY |
| *R. capensis* | South Africa | 30.01.03RC1(HH) | Stoffberg *et al.* 2010 | FJ171195 | THY |
| *R. clivosus* | Tanzania | FMNH-151424 | Zhou *et al*. 2009 | EU436674 | Cytochrome b |
| *R. clivosus* | South Africa | 18.12.02RCL1(SM) | Stoffberg *et al.* 2010 | FJ171196 | THY |
| *R. d. damarensis** | South Africa | TM 48040 | This study | KF683220 | Cytochrome b |
| *R. damarensis* | South Africa | 170109ORC8 | This study | KF683235 | Cytochrome b |
| *R. damarensis* | South Africa | 170109ORC4 | This study | KF683235 | Cytochrome b |
| *R. damarensis** | South Africa | 16112009R?1OR | This study | KF683235, KF683277 | Cytochrome b, THY |
| *R. damarensis** | South Africa | 16112009R?2OR | This study | KF683240 | Cytochrome b |
| *R. damarensis** | South Africa | 16112009R?3OR | This study | KF683235 | Cytochrome b |
| *R. damarensis* | South Africa | 16112009R?4OR | This study | KF683240 | Cytochrome b |
| *R. damarensis** | South Africa | 16112009R?6OR | This study | KF683240 | Cytochrome b |
| *R. damarensis* | South Africa | 16112009R?15OR | This study | KF683248 | Cytochrome b |
| *R. damarensis* | South Africa | 16112009R?16OR | This study | KF683240 | Cytochrome b |
| *R. damarensis** | South Africa | 16112009R?7OR | This study | KF683235, KF683279 | Cytochrome b, THY |
| *R. damarensis* | South Africa | 16112009R?13OR | This study | KF683235, KF683265 | Cytochrome b, THY |
| *R. damarensis* | South Africa | 16112009R?14OR | This study | KF683236, KF683280 | Cytochrome b, THY |
| *R. damarensis** | South Africa | 161109R?10OR | This study | KF683240, KF683277 | Cytochrome b, THY |
| *R. damarensis** | South Africa | 16112009R?9OR | This study | KF683241 | Cytochrome b |
| *R. damarensis** | South Africa | 18112009Rca?2GH | This study | KF683233, KF683274 | Cytochrome b, THY |
| *R. damarensis* | South Africa | 18112009Rca?9GH | This study | KF683237, KF683270 | Cytochrome b, THY |
| *R. damarensis* | South Africa | 18112009Rca?10GH | This study | KF683238, KF683270 | Cytochrome b, THY |
| *R. damarensis* | South Africa | 16112009R?11OR | This study | KF683235, KF683272 | Cytochrome b, THY |
| *R. damarensis** | South Africa | 16112009R?5OR | This study | KF683240 | Cytochrome b |
| *R. damarensis* | South Africa | 16112009R?12OR | This study | KF683240, KF683265 | Cytochrome b, THY |
| *R. damarensis* | South Africa | 16112009R?17OR | This study | KF683240, KF683265 | Cytochrome b, THY |
| *R. damarensis** | South Africa | 16112009R?8OR | This study | KF683239, KF683282 | Cytochrome b, THY |
| *R. damarensis* | South Africa | 23072011Rda02RVM | This study | KF683242, KF683265 | Cytochrome b, THY |
| *R. damarensis* | South Africa | 23072011Rda04RVM | This study | KF683243. KF683265 | Cytochrome b, THY |
| *R. damarensis* | South Africa | 23072011Rda06RVM | This study | KF683244, KF683283 | Cytochrome b, THY |
| *R. damarensis* | South Africa | 21072011Rda03SF | This study | KF683245, KF683265 | Cytochrome b, THY |
| *R. damarensis* | South Africa | 21072011Rda04SF | This study | KF683246, KF683280 | Cytochrome b, THY |
| *R. damarensis* | South Africa | 14012010R?14UN | This study | KF683247 | Cytochrome b |
| *R. damarensis** | South Africa | 18112009Rca?3GH | This study | KF683279 | THY |
| *R. damarensis** | South Africa | 18112009Rca?1GH | This study | KF683278 | THY |
| *R. damarensis** | South Africa | 18112009Rca?5GH | This study | KF683281 | THY |
| *R. damarensis* | South Africa | 16112009R?7OR | This study | KF683235 | Cytochrome b |
| *R. damarensis* | South Africa | 23072011Rda07RVM | This study | KF683265 | THY |
| *R. damarensis* | South Africa | 23072011Rda08RVM | This study | KF683284 | THY |
| *R. damarensis** | South Africa | 14012010R?20UN | This study | KF683274 | THY |
| *R. damarensis** | South Africa | 14012010R?22UN | This study | KF683285 | THY |
| *R. damarensis* | Namibia | 08042010R?17ARN | This study | KF683226 | Cytochrome b |
| *R. damarensis* | Namibia | 08042010R?01ARN | This study | KF683225, KF683265 | Cytochrome b, THY |
| *R. damarensis* | Namibia | 08042010R?03ARN | This study | KF683225, KF683265 | Cytochrome b, THY |
| *R. damarensis* | Namibia | 08042010R?08ARN | This study | KF683225, KF683265 | Cytochrome b, THY |
| *R. damarensis* | Namibia | 08042010R?04ARN | This study | KF683226, KF683266 | Cytochrome b, THY |
| *R. damarensis* | Namibia | 13042010Rda01DAN | This study | KF683227, KF683267 | Cytochrome b, THY |
| *R. damarensis* | Namibia | 08042010R?01MAC | This study | KF683228, KF683269 | Cytochrome b, THY |
| *R. damarensis* | Namibia | 08042010R?02MAC | This study | KF683228, KF683265 | Cytochrome b, THY |
| *R. damarensis* | Namibia | 10042010R?01NOO | This study | KF683229, KF683265 | Cytochrome b, THY |
| *R. damarensis* | Namibia | 10042010R?02NOO | This study | KF683229, KF683272 | Cytochrome b, THY |
| *R. damarensis* | Namibia | 11042010R?01WG | This study | KF683230 | Cytochrome b, THY |
| *R. damarensis* | Namibia | 11042010R?02WG | This study | KF683231, KF683265 | Cytochrome b, THY |
| *R. damarensis* | Namibia | 28072011Rca?05WG | This study | KF683232, KF683265 | Cytochrome b, THY |
| *R. damarensis* | South Africa | 18112009Rca?6GH | This study | KF683234, KF683275 | Cytochrome b, THY |
| *R. damarensis* | South Africa | 18112009Rca?7GH | This study | KF683235, KF683276 | Cytochrome b, THY |
| *R. damarensis* | South Africa | 18112009Rca?8GH | This study | KF683236, KF683265 | Cytochrome b, THY |
| *R. damarensis* | Namibia | 08042010R?06ARN | This study | KF683265 | THY |
| *R. damarensis* | Namibia | 08042010R?07ARN | This study | KF683265 | THY |
| *R. damarensis* | Namibia | 08042010R?04MAC | This study | KF683270 | THY |
| *R. damarensis* | Namibia | 08042010R?06MAC | This study | KF683271 | THY |
| *R. damarensis* | Namibia | 28072011Rca?03WG | This study | KF683265 | THY |
| *R. damarensis** | South Africa | 18112009Rca?4GH | This study | KF683274 | THY |
| *R. damarensis* | South Africa | 14042010RdaGHB | This study | KF683268 | THY |
| *R. darlingi** | Namibia | TM 9474 | This study | KF683224 | Cytochrome b |
| *R. d. barbetonensis** | South Africa | TM 2476 | This study | KF683217 | Cytochrome b |
| *R. darlingi** | South Africa | TM 48018 | This study | KF683218; KF683252 | Cytochrome b, THY |
| *R. darlingi** | South Africa | TM 48019 | This study | KF683218; KF683252 | Cytochrome b, THY |
| *R. darlingi** | South Africa | TM 48020 | This study | KF683219; KF683253 | Cytochrome b, THY |
| *R. darlingi** | South Africa | TM 48021 | This study | KF683219; KF683254 | Cytochrome b, THY |
| *R. darlingi** | South Africa | TM 47765 | This study | KF683219; KF683255 | Cytochrome b, THY |
| *R. darlingi** | Swaziland | TM 47742 | This study | KF683257 | THY |
| *R. darlingi** | South Africa | TM 47947 | This study | KF683256 | THY |
| *R. darlingi* | Zimbabwe | 21102011R_02MT | This study | KF683223, KF683263 | Cytochrome b, THY |
|  |  |  |  |  |  |
| *R. darlingi* | Zimbabwe | 08062012R??02MTP | This study | KF683264 | THY |
| *R. darlingi* | Zimbabwe | 08062012R??03MTP | This study | KF683264 | THY |
| *R. darlingi* | Zimbabwe | 06062012R?01MTP | This study | KF683262 | THY |
| *R. darlingi* | Swaziland | DM5821 | Zhou *et al*. 2009 | EU436675 | Cytochrome b |
| *R. darlingi* | South Africa | 15042012Rda01EH | This study | KF683221, KF683258 | Cytochrome b, THY |
| *R. darlingi* | South Africa | 15042012Rda02EH | This study | KF683222, KF683258 | Cytochrome b, THY |
| *R. darlingi* | South Africa | 15042012Rda01EH | This study | KF683221 | Cytochrome b |
| *R. darlingi* | South Africa | 15042012Rda02EH | This study |  |  |
| *R. darlingi* | South Africa | 15042012Rda03EH | This study | KF683259 | THY |
| *R. darlingi* | South Africa | 13042012Rda01SUD | This study | KF683260 | THY |
| *R. darlingi* | Zimbabwe | 19062012Rda02CHK | This study | KF683261 | THY |
| *R. darlingi* | South Africa | DSJ9 | Eick *et al.* 2005 | AJ865673 | THY |
| *R. darlingi* | South Africa | 26.07.02RD(MC) | Stoffberg *et al.* 2010 | FJ171197 | THY |
| *R. denti* | Namibia | 11042010Rde?01WG | This study | KF683249, KF683273 | Cytochrome b, THY |
| *R. denti* | South Africa | 20.07.04RDNT4(KGB) | Stoffberg *et al.* 2010 | FJ171198 | THY |
| *R. eloquens* | Kenya | CM-97948 | Zhou *et al.* 2009 | EU436677 | Cytochrome b |
| *R. euryale* | Spain | EBD-24814 | Zhou *et al.* 2009 | EU436671 | Cytochrome b |
| *R. ferrumequinum* | Spain | EBD-24818 | Zhou *et al.* 2009 | EU436673 | Cytochrome b |
| *R. ferrumequinum* | China | 68 Zhang SY | Li *et al.* 2006 | DQ297575 | Cytochrome b |
| *R. formosae* | Taiwan | 2005.65.40 | Stoffberg *et al.* 2010 | FJ171199 | THY |
| *R. fumigatus* | Namibia | 13042010Rfu08DAN | This study | KF683286 | THY |
| *R. fumigatus* | Kenya | CM-97951 | Zhou *et al.* 2009 | EU436678 | Cytochrome b |
| *R. fumigatus* | South Africa | 4.08.02RF1(LG) | Stoffberg *et al.* 2010 | FJ171200 | THY |
| *R. fumigatus* | Tanzania | WTS1533 | Stoffberg *et al.* 2010 | FJ171201 | THY |
| *R. fumigatus* | South Africa | 31.7.02Rf (TH) | Eick *et al.* 2005 | AJ865674 | THY |
| *R. hildebrandti* | Tanzania | FMNH-151422 | Zhou *et al.* 2009 | EU436676 | Cytochrome b |
| *R. hildebrandti* | Tanzania | WTS2562 | Stoffberg *et al.* 2010 | FJ171203 | THY |
| *R. hildebrandti* | South Africa | 18.12.02RH1(SM) | Stoffberg *et al.* 2010 | FJ171202 | THY |
| *R. hipposideros* | UK | DRH Jones G | Li *et al.* 2006 | DQ297586 | Cytochrome b |
| *R. hipposideros* | Greece | PB875 | Stoffberg *et al.* 2010 | FJ171204 | THY |
| *R. landeri* | Kenya | CM-97952 | Zhou *et al.* 2009 | EU436668 | Cytochrome b |
| *R. landeri* | South Africa | 2.08.02RL1(TK) | Stoffberg *et al.* 2010 | FJ171205 | THY |
| *R. lepidus* | Myanmar | MW2 | Stoffberg *et al.* 2010 | FJ171206 | THY |
| *R. luctus* | China | T234 Zhang SY | Li *et al.* 2006 | DQ297596 | Cytochrome b |
| *R. maclaudi* | Uganda | WTS889 | Stoffberg *et al.* 2010 | FJ171207 | THY |
| *R. macrotis* | China | B00011, Zhang S.Y. | Cui *et al.* 2007 | EF517312 | Cytochrome b |
| *R. marshalli* | Myanmar | TA3 | Stoffberg *et al.* 2010 | FJ171210 | THY |
| *R. megaphyllus* | Australia | EBU12131 | Stoffberg *et al.* 2010 | FJ171211 | THY |
| *R. megaphyllus* | Australia | EBU12131 | Stoffberg *et al.* 2010 | FJ171211 | THY |
| *R. mehelyi* | Spain | EBD-24813 | Zhou *et al.* 2009 | EU436672 | Cytochrome b |
| *R. monoceros* | Taiwan | 2005.65.57 | Stoffberg *et al.* 2010 | FJ171212 | THY |
| *R. pearsonii* | China | B00014, Zhang S.Y. | Cui *et al.* 2007 | EF517310 | Cytochrome b |
| *R. pearsonii* | Myanmar | SH8 | Stoffberg *et al.* 2010 | FJ171213 | THY |
| *R. pusillus* | China | T245 Zhang SY | Li *et al.* 2006 | DQ297597 | Cytochrome b |
| *R. pusillus* | Myanmar | SH15 | Stoffberg *et al.* 2010 | FJ171214 | THY |
| *R. ruwenzorii* | Uganda | FMNH-144309 | Zhou *et al.* 2009 | EU436679 | Cytochrome b |
| *R. simulator* | Zimbabwe | 24102011RSi01CC | This study | KF683250 | Cytochrome b |
| *R. simulator* | South Africa | 12.08.03Runk1(SM2) | Stoffberg *et al.* 2010 | FJ171215 | THY |
| *R. simulator* | Tanzania | WTS2561 | Stoffberg *et al.* 2010 | FJ171216 | THY |
| *R. sinicus* | China | A066 | Cui *et al.* 2007 | EF517304 | Cytochrome b |
| *R. sinicus* | Nepal | 98.5.6 | Stoffberg *et al.* 2010 | FJ171217 | THY |
| *R. swinnyi* | South Africa | 15042012Rsw01EH | This study | KF683251 | Cytochrome b |
| *R. swinnyi* | South Africa | 23.07.04RSW4(KSM) | Stoffberg *et al.* 2010 | FJ171218 | THY |
| *R. thomasi* | Myanmar | SH13 | Stoffberg *et al.* 2010 | FJ171219 | THY |

^1^The species are reported in alphabetical order and detail the collection locality (where known), the tissue identification number, the source of the sequence (when obtained from GenBank, authors of the publication or, for unpublished sequences, authors of the GenBank entry are given), the corresponding accession number and the sequenced gene.

^2^ *R. damarensis* = *R. darlingi* reclassified in this study on the basis of genetic analyses.

^3^Individuals which were included in the phenotypic analyses are marked with an asterisks (*).

**Table S4.** Primers used to amplify short fragments of the cytochrome b gene designed for this study.

| Name | Sequence (5´to 3´) | Length (bp) | Tm (°C) | GC (%) | Product size (bp) |
| --- | --- | --- | --- | --- | --- |
| DaF1-F | CGTTCGTTGACCTAC CCGCCC | 21 | 59.79 | 66.67 | 216 |
| DaR1-R | TGGAGGCTCCGTTGGCATGGA | 21 | 60.18 | 61.90 |  |
| DaF2-F | AGCCGGAATCCCATCAGACGC | 21 | 58.98 | 61.90 | 132 |
| DaR2-R | TCACCCAGTAGGTCGGGGGC | 20 | 59.61 | 70.00 |  |
| DaF3-F | ATCTGCCTGTTCCTACACGT | 20 | 58.73 | 50.00 | 227 |
| DaR3-R | CCGCCTCAGACTCATTCAAC | 20 | 58.64 | 55.00 |  |
| DaF4-F | CAGCCAACCCACTAAACACC | 20 | 59.04 | 55.00 | 200 |
| DaR4-R | TCGAAATGTCATGCTGCGTT | 20 | 58.84 | 45.00 |  |
| DAF5-F | ACACTCACCCGATTCTTCGCCCT | 23 | 58.70 | 56.50 | 222 |
| DAR5-R | AGTAGGTCGGGGGCAAATAG | 20 | 53.70 | 55.00 |  |

^1^Primer name is followed by the sequence, primer length, the melting temperature, GC-content and average size of the amplified product.

**Table S5.** Principal component loadings for the first three components extracted from the 24 skull and external measurements.

|  | Component 1 | | Component 2 | Component 3 |
| --- | --- | --- | --- | --- |
| ALSW | | -0.88 | -0.15 | 0.06 |
| AMSW | | -0.58 | -0.31 | 0.07 |
| UCM3L | | -0.92 | -0.29 | 0.02 |
| IOW | | -0.58 | 0.5 | 0.16 |
| M3M3W | | -0.93 | -0.17 | -0.13 |
| MW | | -0.79 | -0.43 | 0.06 |
| PL | | 0.15 | -0.62 | 0.51 |
| SL | | -0.93 | -0.26 | 0.08 |
| ZW | | -0.94 | -0.24 | -0.13 |
| ML | | -0.94 | -0.21 | -0.02 |
| LCM3L | | -0.93 | -0.25 | -0.03 |
| FA | | -0.91 | 0.08 | 0 |
| M-III | | -0.9 | -0.1 | 0.1 |
| P1-III | | -0.88 | 0.33 | -0.01 |
| P2-III | | -0.86 | 0.27 | 0.08 |
| M-IV | | -0.95 | -0.01 | 0.03 |
| P1-IV | | -0.91 | 0.32 | 0.05 |
| P2-IV | | -0.81 | 0.17 | 0.31 |
| M-V | | -0.93 | 0.16 | 0.1 |
| P1-V | | -0.81 | 0.3 | 0.2 |
| P2-V | | -0.79 | 0.23 | -0.06 |
| TB | | -0.85 | 0.29 | -0.08 |
| TL | | -0.57 | 0 | -0.62 |
| EL | | -0.48 | -0.33 | -0.43 |
| Eigenvalue | | 16.28 | 1.97 | 1.09 |
| Cumulative Eigen value | | 16.28 | 18.25 | 19.34 |
| Cumulative % variance explained | | 67.83 | 76.04 | 80.59 |

^1^Measurement abbreviations are given in Table S1, Appendix S1.

**Table S6.** The means ± standard deviations, ranges and sample sizes of bacula measurements (in mm) for several species of southern African rhinolophids.

|  | *R. blasii* | *R. capensis* | *R. clivosus* | *R. d. damarensis* | *R. d. darlingi* |
| --- | --- | --- | --- | --- | --- |
| ^1^GBL | 2.8 ± 0.1  2.8-2.9  2 | 2.6 ± 0.3  2.3-2.8  2 | 3.7 ± 0.5  2.9-4.0  5 | 3.3 ± 0.25  2.9-3.9  11 | 2.6 ± 0.1  2.4-2.7  3 |
| GW | 0.2 ± 0.03  0.1-0.2  2 | 0.2 ± 0.04  0.2-0.2  2 | 0.8 ± 0.1  0.6-0.9  6 | 0.4 ± 0.1  0.3-0.4  11 | 0.3 ± 0.04  0.3-0.3  3 |
| WB-d | 0.8 ± 0.04  0.8-0.8  2 | 1.0 ± 0.0  1.0-1.0  1 | 1.2 ± 0.3  0.7-1.5  5 | 1.3 ± 0.1  1.1-1.4  11 | 0.9 ± 0.0  0.9-0.9  3 |
| WB-l | 0.9 ± 0.04 0.9-0.9  2 | 0.8 ± 0.0  0.5-0.9  2 | 0.9 ± 0.3  0.6-1.1  5 | 1.2 ± 0.1  0.7-1.3  11 | 0.9 ± 0.0  0.9-0.9  3 |
| LTW | 0.1 ± 0.0  0.07-0.09  2 | 0.1 ± 0.1  0.05-0.14  2 | 1.0 ± 0.2  0.8-1.4  6 | 0.7 ± 0.3  0.2-1.2  11 | 0.4 ± 0.1  0.3-0.5  3 |
| NW | 0.1 ± 0.04  0.09-0.14  2 | 0.1 ± 0.04  0.1-0.2  2 | 0.3 ± 0.1  0.2-0.5  6 | 0.3 ± 0.05  0.2-0.4  11 | 0.2 ± 0.03  0.18-0.23  3 |
| LTN | 0.2 ± 0.03  0.2-0.3  2 | 0.4 ± 0.04  0.36-0.41  2 | 2.3 ± 0.32  1.9-2.6  6 | 1.8 ± 0.2  1.4-2.1  11 | 1.5 ± 0.1  1.4-1.6  3 |
| BIL | 0.3 ± 0.1  0.2-0.3  2 | 0.3 ± 0.04  0.2-0.3  2 | 0.4 ± 0.1  0.3-0.6  5 | 0.7 ± 0.1  0.3-0.8  11 | 0.3 ± 0.1  0.3-0.4  3 |
| BH | 0.8 ± 0.3  0.7-0.9  2 | 0.6 ± 0.2  0.4-0.7  2 | 1.0 ± 0.3  0.5-1.2  5 | 1.0 ± 0.1  0.7-1.1  11 | 0.8 ± 0.1  0.7 0.9  3 |

^1^ Key to the abbreviations are given in the text.

**Appendix S2: Supplementary methods**

*Phylogenetic reconstruction and dating*

*DNA extraction of museum specimens.* DNA was extracted from dried skin samples taken from the museum specimens (TM9474 and TM2476) using a QIAamp DNA Micro Kit (Qiagen) following the manufacturers’ instructions with a number of modifications. Prior to digestion, the small tissue samples were first washed twice with 1 ml 99% ethanol and once with 1 ml 70% ethanol. At each step the samples were vortexed for 2 minutes. The tissue was then soaked in double-distilled sterilised water and incubated for 48 hours at 50°C. After removing the water, the samples were then digested at 56°C for 24 hours in 180 µl buffer ATL and 40 µl proteinase K. The manufacturer’s protocol was then followed repeating the elution step twice with 60 µl and 40 µl to maximise the DNA yield from the samples. The resulting DNA templates had concentrations of 13.9 ng/µl (TM9474) and 17.5 ng/µl (TM2476) versus 21.0 – 67.0 ng/µl from the fresh tissue samples (Nanodrop Spectrophotometer, Thermo Fisher Scientific Inc.).

*PCR conditions for amplifying cyt b.* Optimal concentrations and temperature conditions for PCR amplification of the cyt b gene were ascertained by running gradients and comparing the resulting PCR products on 1% agarose gel under UV. Total PCR reaction volume was 30 μl containing 1 x buffer (10x with loading dye), 1.4 mM µl MgCl2, 0.2 mM dNTP´s, 0.5 mM of each primer, 0.05 mM Taq (5U KAPATaq) and 4.5 µl template DNA. PCR was carried out on a GeneAmp 2720 Thermocycler (Applied Biosystems) under the following conditions: 39 cycles of 94°C for 45 sec, 50°C for 30 sec, 72°C for 70 sec, followed by one cycle of 94°C for 30 sec, 50°C for 30 sec and, 72°C for 310 sec and finally cooled down to 4°C.

The standard PCR conditions described above successfully amplified a >950 bp fragment of cyt b in fresh tissue samples; however we were not able to amplify the marker using these primers in the museum samples, most likely due the degraded nature of the extracted DNA.. Following Weber *et al.* (2000, 2004) we therefore used a method of template reconstruction followed by cold-start PCR using a set of five primers designed to target short, overlapping fragments of the cyt b gene. The first step in this process used “primerless” PCR (Stemmer, 1994 Cotterill, 2006) where standard PCR ingredients are used to extend fragmented DNA pieces in the absence of primers; this is because the fragments themselves act as primers. The reconstructed DNA from the primerless PCR was then used as the template in a second Cold-Start PCR with a set of primers added (Table S3). The following master mix was used in both PCR reactions: a 50 µl reaction containing 0.5X buffer (10x) with loading dye, 0.375 mM MgCl2, 8x10^-8^ mM dNTP´s, 0.5 mM of each primer, 0.0125 mM Taq (5U KAPATaq) and 3.0 µl template DNA. The primerless PCR was carried out on a GeneAmp 2720 Thermocycler under the following conditions: 94°C for 90 sec (initial denaturation), followed by 35 cycles of 94°C for 60 sec (denaturation), 65°C for 60 sec (primer annealing) and 72°C for 90 sec (elongation), with a final step at 72°C for 60 sec (final elongation). The Cold-Start PCR for the second reaction, with PCR primers added, entailed a first step at 95°C for 3 min, followed by 5 cycles of 95°C for 30 s, 45°C for 45 s and 72°C for 60 s. The subsequent 35 cycles started at 95°C for 30 s, 51°C for 45 s and 72°C for 90 s. PCR ended with a final elongation step at 72°C for 5 min. PCRs of museum samples were performed on a slow-ramp Hybaid Thermal Reactor (Hybaid Limited, UK). As before, PCR products were purified using a Wizard SV Gel and PCR Clean-up System (Promega) and sequenced in both directions using BigDye 3.1 chemistry on an ABI 3730 XL DNA Analyzer (Applied Biosystems).

*PCR conditions amplifying THY.* Total reaction volume was 50 µl containing 0.6 mM buffer (10X with loading dye), 1.5 mM MgCl2, 0.12 mM dNTP´s, 0.3 mM of each primer, 0.03 mM Taq (5U KAPATaq) and 5.0 µl template DNA. A PCR was run on the samples in a Thermocycler (2720 Thermal Cycler Applied Biosystems) under the following thermal conditions: 94°C for 120 sec followed by 34 cycles of 94°C for 30 sec, 53°C for 60 sec and 72°C for 30 sec followed by one cycle at 72°C for 600 min at a cool down to 4°C. PCR products were purified using a Wizard SV Gel and PCR Clean-up System (Promega) and sequenced in both directions using BigDye 3.1 chemistry on an ABI 3730 XL DNA Analyzer (Applied Biosystems).

*Analyses settings for Maximum Parsimony and Maximum Likelihood analyses for cytochrome b and THY.* All sequences entered the analyses as single taxa and all characters entered the analyses with equal weighting. Gaps were treated as missing data. MP analysis was conducted on informative positions only. We conducted heuristic searches with 10 replicates. The starting tree was obtained via stepwise addition of taxa which were added randomly. One tree was held after each addition. We chose tree-bisection-reconnection method as branch-swapping (TBR) algorithm. To assess reliability of clades the number of bootstrap replicates was set to 1000 with an adjusted approximation limit of 0.7 to decrease computation time. To control for effects of multiple substitutions, distance corrections can be calculated and applied as best fit substitution models for the ML analysis. To choose the model we entered data sets into the software ModelTest version 3.7 (Posada & Crandall, 1998) to find the best model of nucleotide evolution for each data set. The Akaike information criterion and the hierarchical likelihood ratio test resulted in identical best fit models. The model settings were transferred into PAUP*. The Transitional Model TVM+I+G (Posada, 2003) model best fitted the data for cyt b. This model accounts for variable base frequencies, variable transversion rates and equal transition rates. Result was saved displayed as 50% majority-rule consensus tree. The K81uf+G substitution model best fitted to the THY dataset accounting for equal transversions rates.

*Bayesian phylogenetic analysis.* Tree reconstruction using Bayesian inference was carried out in MrBayes 3.1.2 (Ronquist & Huelsenbeck, 2003). The two data sets were first analysed separately and then sequences were concatenated for a subset of samples and analysed together. The concatenated ‘cyt b + THY’ data set was analysed using a partitioned analysis (sequence data and model parameters) to allow independent convergence on model parameters for each gene fragment (Ronquist & Huelsenbeck 2003). We implemented the general time reversible model (GTR; Tavaré 1986) with gamma distributed rate variation and a proportion of invariant sites i.e. GTR + I + G. For all analyses random starting trees were used together with default priors. A total of five million generations were run using four unheated Markov chains sampling the distribution every 100 generations; convergence of the MCMC chains was determined when the standard deviation of split frequencies was <0.01 and the potential scale reduction factor was 1.0 for all parameters (Ronquist & Huelsenbeck 2003). Convergence for all runs was also assessed in Tracer v1.5 (Drummond & Rambaut 2007) and the final analysis was based on a burn-in of 25%. Reproducibility together with effective mixing of the MCMC was established by multiple analysis runs from independent starting points. Results are summarised as the majority rule consensus of all the trees sampled, using *Hipposideros* spp. to root the resulting trees.

*Molecular dating.* We estimated the time to most recent common ancestor (TMRCA) for a number of clades of interest using BEAST v1.7.4 (Drummond & Rambaut 2007). We used the HKY model with empirical base frequencies and the dating was performed using a relaxed uncorrelated lognormal molecular clock together with the Yule speciation process tree prior (Drummond et al. 2006) The analysis was performed on the cyt b data set and calibrated using lognormal tree priors with (i) an upper limit of 5.3 Ma and a lower limit of 1.8 Ma based on fossil dates for *R. ferrumequinum*, described from deposits in Poland (Woloszyn 1987), and (ii) an upper limit of 55 Ma and a lower limit of 37 Ma for the split between the Rhinolophidae and the Hipposideridae, following Teeling et al. (2003) and Eick et al. (2005). The phylogenetic analysis revealed that *R. ferrumequinum* is paraphyletic with *R. clivosus*; the lineage was therefore calibrated at the root of the *ferrumequinum/clivosus* clade. The MCMC chain was run for 25 million generations, with parameters logged every 1000 generations. The results were checked in Tracer v1.5 and the effective sample size (ESS) values were > 200 for all parameters estimated suggesting that the MCMC run had reached stationarity. Reproducibility was established by multiple analysis runs from independent starting points and the topologies and posterior probabilities were compared for consistency. The resulting trees were collated and analysed in TreeAnnotator v1.7.4 using mean heights and a 10% burn-in.

*Parameter settings for MaxEnt Analyses*

MaxEnt allocates a probability of occurrence, based on presence data, to each cell in the study area and the sum of this probability must be equal to one. Therefore, we presented the model predictions as graded probabilities for each grid cell ranging from zero (low) to one (high) probability of occurrence and imported the output format to ArcGIS 10. Prior to running the models, the parameters were set as follows: regularisation multiplier to 1, maximum number of background points (pixels) to 10,000, replicates to 10, replicated run type to cross-validate, maximum iterations to 500, and conversion threshold to 10-5. The environmental variables ‘features’ selection was performed automatically following the default rules set according to the number of presence records. MaxEnt allocates a probability of occurrence to each cell in the study area and the sum of this probability must be equal to one. Therefore, we presented the model predictions as graded probabilities for each grid cell ranging from zero (low) to one (high) probability of occurrence and imported the output format to ArcGIS 10.

**Appendix S3: Supplementary results**

*Principal component analyses*

PCA extracted 24 factors of which the first three explained 80% of the variance. The first three factors were also the only factors with an Eigenvalue ≥ 1 (Table S4). All variables, most of them associated with size (e.g. forearm, skull length, length of digits), had highest loadings on Factor 1. Only palate length and tail length loaded higher on Factor 2 and Factor 3, respectively (Table S4). Palatal length was important on all three factors. On the first factor it was the only measurement with a positive sign. The majority of the other measurements with negative signs on the first factor had high scores, the highest being for zygomatic width, mandible length and length of metacarpal of 4^th^ digit. On the second and third axes interorbital width and tail length loaded highest respectively.

**Supplementary References**

Cui J, Han N, Streicker D, Li G, Tang X, et al. (2007) Evolutionary relationships between bat

coronaviruses and their hosts. Emerg Infect Diseases 13: 1526-1532.

Drummond AJ, Rambaut A (2007) BEAST: Bayesian evolutionary analysis by sampling

trees. BMC Evol Biol **7:** 214.

Drummond AJ, Ho SYW, Phillips MJ, Rambaut A (2006) Relaxed phylogenetics and dating

with confidence. PLoS Biol 4(5): e88. doi:10.1371/journal.pbio.0040088.

Eick GN, Jacobs DS, Matthee CA (2005) A nuclear DNA phylogenetic perspective

on the evolution of echolocation and historical biogeography of extant bats (Chiroptera). Mol Biol Evol 22: 1869-1886.

Guillen-Servent A, Francis CM (2006) A new species of bat of the *Hipposideros bicolor*

group (Chiroptera: Hipposideridae) from Central Laos, with evidence of convergent evolution with Sundaic taxa. Acta chiropterol 8: 39-61.

Li S-H, Yeung CKL, Feinstein J, Han L, Le MH, et al. (2009) Sailing through the Late

Pleistocene: unusual historical demography of an East Asian endemic, the Chinese Hwamei (*Leucodioptron canorum canorum*), during the last glacial period. Mol Ecol 18: 622-633.

Niu H, Wang N, Zhao L, Liu J (2007) Distribution and underground habitats of cave-

dwelling bats in China. Anim Cons 10: 470-477.

Posada D, Crandall KA (1998) Modeltest: testing the model of DNA substitution.

Bioinformatics 14: 817-818.

Ronquist F, Huelsenbeck JP (2003) MrBayes 3: Bayesian phylogenetic inference under

mixed models. Bioinformatics 19: 1572-1574.

Stoffberg S, Jacobs DS, Matthee CA (2011) The divergence of echolocation

frequency in Horseshoe Bats: moth hearing, body size or habitat? J Mammal Evol 18: 117–129. DOI 10.1007/s10914-011-9158-x.

Sun K, Feng J, Jin L, Liu Y, Shi L, et al. (2009) Structure, DNA sequence variation and

phylogenetic implications of the mitochondrial control region in horseshoe bats. Z Saeuget 74: 130-144.

Tavaré S (1986) Some probabilistic and statistical problems in the analysis of DNA

sequences. Lectures Math. Life Sci 17: 57-86.

Teeling EC, Madsen O, Murphy WJ, Springer MS, O’Brien SJ (2003) Nuclear

gene sequences confirm ancient link between New Zealand’s short-tailed bat and South American noctilionoid bats. Mol Phylogenet Evol 28: 308-319.

Weber DS, Stewart BS, Garza JC, Lehman N (2000) An empirical genetic assessment of the

severity of the northern elephant seal population bottleneck. Curr Biol 10: 1287-1290.

Weber DS, Stewart BS, Lehman N (2004) Genetic consequences of a severe population

bottleneck in the Guadalupe fur seal (*Arctocephalus townsendi*). J Hered 95: 144-153.

Woloszyn BW (1987) Pliocene and Pleistocene bats of Poland. Acta Palaeontol Pol 32: 207–

325.

Zhou ZM, Guillen-Servent A, Lim BK, Eger JL, Wang YX, Jiang, XL (2009) A new species

from southwestern China in the Afro-Palearctic lineage of the Horseshoe bats (*Rhinolophus*). J Mammal 90: 57–73.
