# Supplementary figures and images for: Phenotypic Convergence in Genetically Distinct Lineages of a Rhinolophus Species Complex (Mammalia, Chiroptera)
Source: PLoS One. 2013 Dec 3;8(12):e82614. doi: 10.1371/journal.pone.0082614 (PMC3849494; doi:10.1371/journal.pone.0082614)

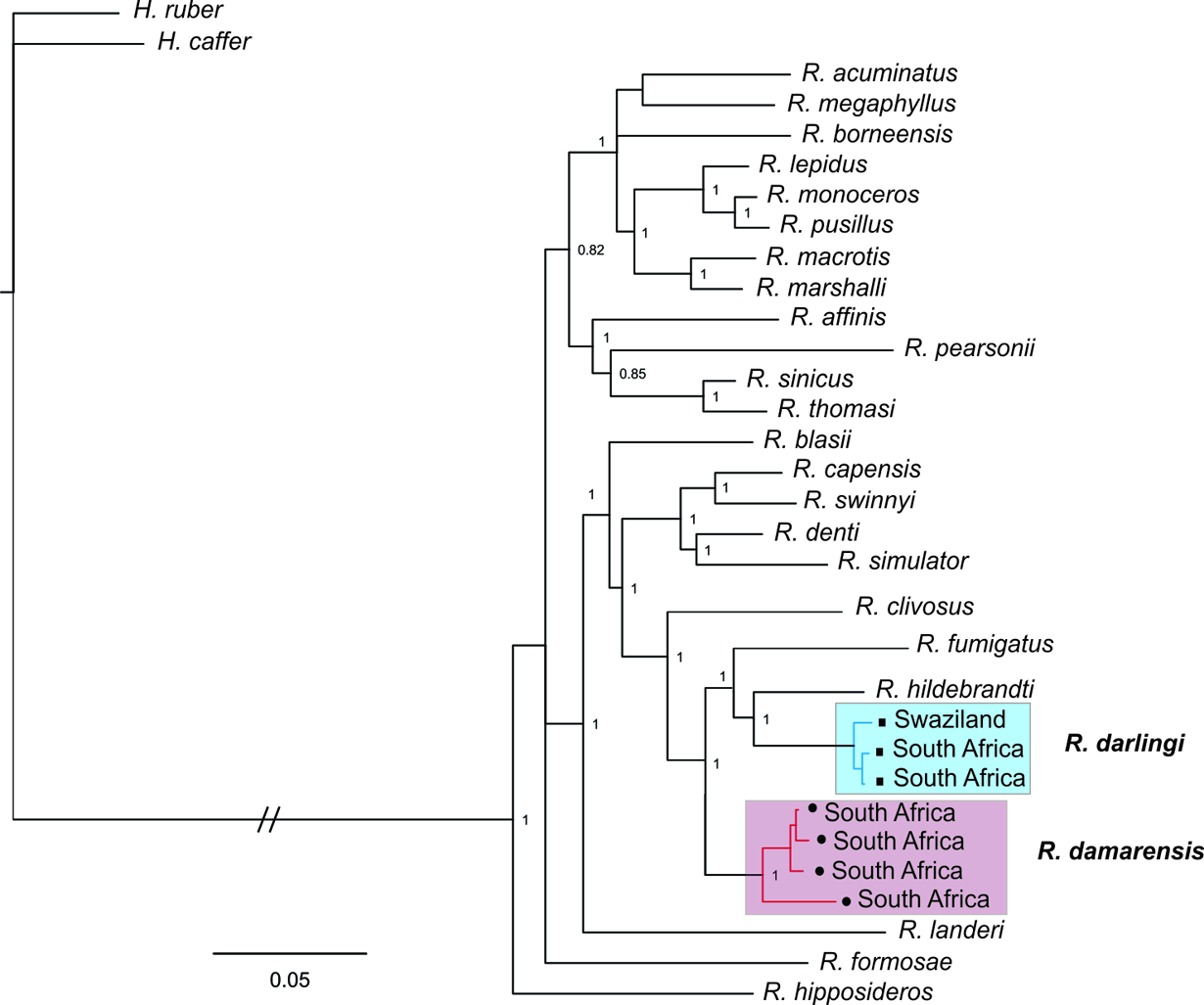

Supplement: Figure S1 — Bayesian consensus topology of Rhinolophus spp. based on a combined analysis of cyt b and THY on a subset of species for which both markers were available. (TIF) [file pone.0082614.s002.tif]
